# Supplementary material for: Population discontinuity in the Paris Basin linked to evidence of the Neolithic decline
Source: Nat Ecol Evol. 2026 Apr 3;10(4):677–88. doi: 10.1038/s41559-026-03027-z (PMC13076197; doi:10.1038/s41559-026-03027-z)
Supplement: Supplementary file 1 — Supplementary Notes 1–7 and Figs. 1.1–7.6. [file 41559_2026_3027_MOESM1_ESM.pdf]

nature ecology & evolution

Supplementary information

https://doi.org/10.1038/s41559-026-03027-z

Population discontinuity in the Paris Basin linked to evidence of the Neolithic decline

In the format provided by the authors and unedited

Supplementary Information

For *Population discontinuity in the Paris Basin linked to evidence of the Neolithic Decline*

Seersholm, Ramsøe, Cao et al.

Supplementary Note 1 - Demographic anomalies in the collective burials from the fourth millennium BC in Northern France and Germany

Philippe Chambon and Laure Salanova

Despite the hundreds of collective burials dated from the fourth millennium BC, very little data is available for demographic analyses. With two main burial periods, the Bury grave provides the ideal conditions to follow a population over a long sequence[12].

At Bury, the mortality profiles for the two burial periods are clearly distinct (Supplementary Fig. 1.1A). The first phase is the most divergent: with the exception of the [1-4] age group, the mortality rates never fall within the expected limits. Infant mortality is—as often in these graves[82]—underrepresented. For the other three age groups, quotients exceed extreme values, even when considering low life expectancy at birth. For Phase 2 on the other hand, the mortality profile complies with the pre-Jennerian scheme from the [5-9] age group upwards.

In other collective burials in Northern France, mortality profiles are not identical from one tomb to another. However, none of these profiles correspond to attritional mortality in a closed and stationary population: there is always excess in one or the other non-adult age groups (Supplementary Fig. 1.1B). Deviation from the reference population is not specific to one category of architecture (Supplementary Fig. 1.1C).

Collective graves from other areas in Europe offer a similar pattern. In Germany, the Galeriegräber from Hessen and Westphalian and the Totenhütten from Saxony and Thuringia show an interruption of burials at the end of the fourth millennium, as recently emphasised[9,10,46]. Again, the demographic data do not correspond to attritional mortality, with overrepresentation of any of the non-adults age groups (Supplementary Fig. 1.1D).

At Bury phase 1, one alternative would be a rapid increase of the population. A second alternative is a crisis that primarily affected individuals before 15 years. Troubled times, such as multiple conflicts, do not fit: skeletons of the age groups in question do not show such deleterious damage. Deaths must have been fast enough to not affect tooth or bone development. A disease that affects children of any age, such as plague, appears a good candidate. Both alternatives lead to the same issue: a demographic crisis is generally the result of a huge increase of the population.

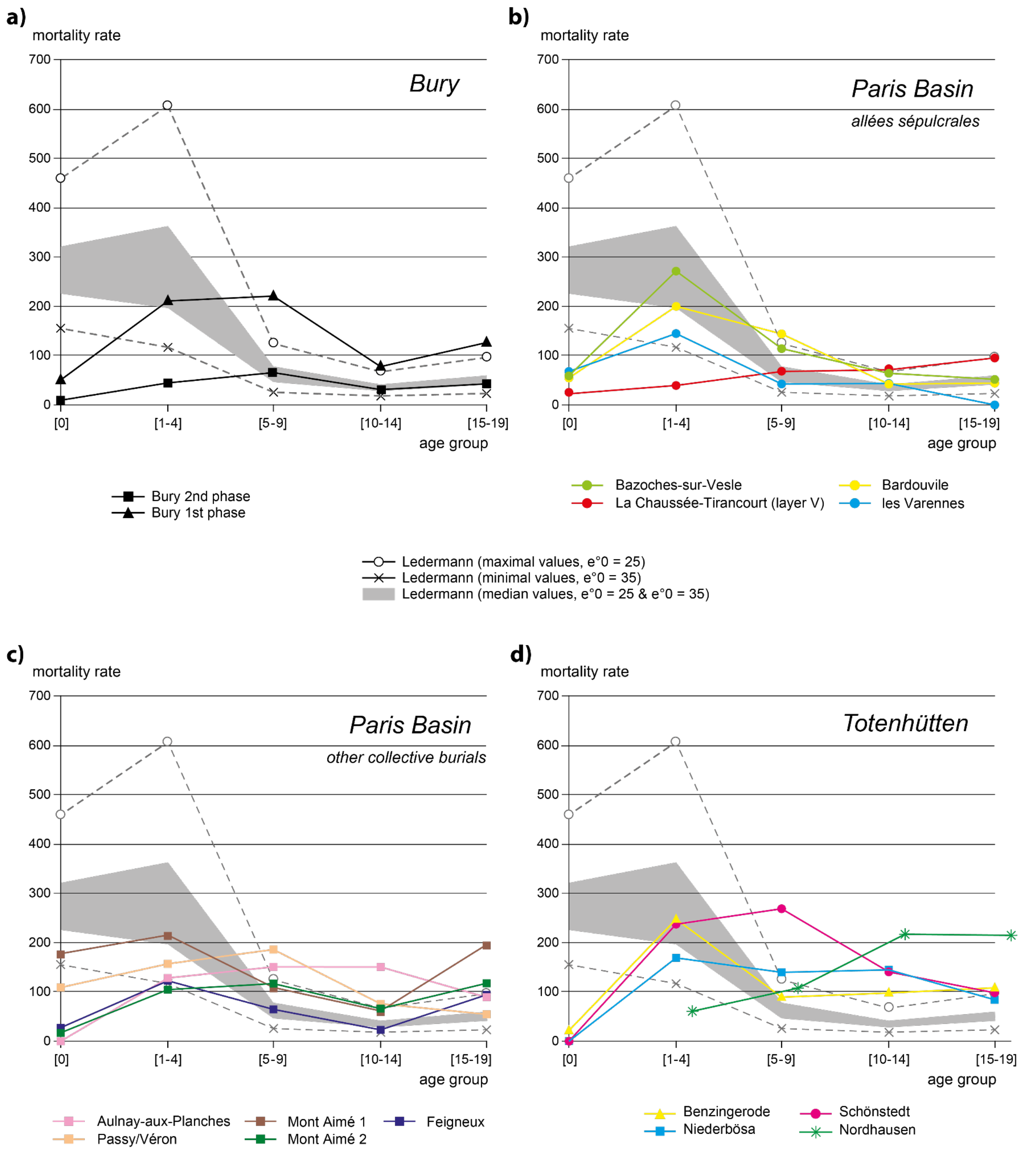

Supplementary Figure 1.1. Demographic profiles of the individuals found in collective graves dated from the fourth millennium BC, limited to non-adults age groups and using reference curves for a stationary population[83,84]. The grey area constricts attritional mortality of stationary populations. **A)** Bury; **B)** allées sépulcrales from Northern France[85–87]; **C)** other collective graves from Northern France[88–91]; **D)** Totenhütten from Saxony and Thuringia[92–94]. The differences to the reference population have been minimised[84].

Supplementary note 1.1 - Morphological sex determination

On the basis of the coxal bone, the sex estimate counted 24 females and 52 males for the entire grave. Due to the lack of well-preserved coxal bones, this estimate is only one third of

the minimum number of individuals over 15 (228). Indicative if not significant, the imbalance affects both phases, 12/29 in phase 1 and 22/24 in phase 2.

Supplementary Note 2 - Population genomics, kinship analysis and plague genetics

Frederik V. Seersholm

Supplementary Note 2.1 - Mixture modelling source groups

AnatolianFarmers: Bon004, I0708, I1098, Tep002, Tep003, Tep004

BellBeaker: I13025, I13028, I5748

CHG: KK1, NEO281, SATP

Early Neolithic France: BUCH2, CB13, GLN246, GLN275, GLN284, GLN285A, GLN308, GLN309, GLN320, I0410, I0412, I0413, I4304, NEO812, R6, mur

IranNeolithic: AH4, GD13a, I1954, NEO816, WC1

Middle Neolithic Iberia:I8134, TOR6, LugarCanto41, I0406

Yamnaya: I0231, I0370, I0438, I0443, RISE547, RISE550

balticCentralEurHG: Donkalnis6, KO1, Latvia_HG2, NEO307, PL_N22, Spiginas1

earlyCentralEurNeolithicLBK: I0025, I0026, I15818, I1904, I2739, Sch72-15

earlySpainHG: Chan, NEO694

russianHG: Latvia_MN2, NEO166, NEO167, NEO170, NEO171, NEO178, NEO179, NEO180, NEO184, NEO186, NEO189, NEO192, NEO193, NEO194, NEO195, NEO197

scandinavianNeolithic: CGG106494, NEO38, NEO43, NEO46, NEO744, NEO757, NEO896, ans003

ukraineHG: NEO270, NEO501, NEO521, NEO524, NEO552, Ukraine_N1

westernHG: Loschbour, PER1150503, R7, SRA62, Villabruna

Supplementary Note 2.2 - Pedigree descriptions

Pedigree 1.A

Generations represented: 5
Number of individuals (sampled): 29
Number of individuals (total): 48
Sampled males/females: 21/8 (72% males)

Consisting of three brothers, and their descendants, this pedigree is the largest of the study, spanning at least five generations. All male individuals belong to chromosome Y haplogroup H2a1. Furthermore, this pedigree stands out because of its elevated levels of runs of homozygosity (RoH) as compared to all other pedigrees in this study (Extended Data Fig. 8), suggesting a high level of inbreeding in this family. No individuals in this group were positive for *Yersinia pestis*.

Pedigree 1.B

Generations represented: 4
Number of individuals (sampled): 13
Number of individuals (total): 23
Sampled males/females: 9/4 (69% males)

The first generation of pedigree 1.B consists of a set of parents (BUR266 and BUR258), and the three brothers BUR232, BUR179 and BUR218 who form an unknown avuncular relationship to BUR266. All males of this pedigree carry the chromosome Y haplogroup I2a1, except for BUR185, who carries H2a1. It could imply that BUR185's father has some genetic relationship to pedigree group 1.A, however this link has not been detected genetically - it could be that the relatives linking this individual with pedigree group 1.A either were not sampled or were not buried in the grave. However, another possibility is that BUR185 has no relation to pedigree group 1.A, and that he was buried in the grave either due to having grandparents in group 1.B, or a societal relationship such as adoption or fostering33, which is untraceable by DNA alone.

Plague was detected in BUR218.

Pedigree 1.C

Generations represented: 4
Number of individuals (sampled): 6
Number of individuals (total): 13
Sampled males/females: 4/2 (67% males)

This pedigree consists of two brothers (BUR321 and BUR 202), their sons (BUR269 and BUR225), and the granddaughter (BUR209) of BUR202. Additionally, there is an uncertain relationship between BUR202 and BUR327, which is difficult to determine because of low coverage. However, their shared mitochondrial haplogroup, combined with their sex (both are women) and their high IBD sharing suggests that

they are mother and daughter. All males of pedigree 1.C carry the chromosome Y haplogroup H2.

Pedigree 1.D

Generations represented: 2
Number of individuals (sampled): 2
Number of individuals (total): 4
Sampled males/females: 2/0 (100% males)

Pedigree 1.D comprises the two brothers BUR290 and BUR341 and their unsampled parents. Both brothers carry the I2a1 chromosome Y haplogroup.

Pedigree 1.E

Generations represented: 2
Number of individuals (sampled): 2
Number of individuals (total): 4
Sampled males/females: 2/0 (100% males)

Pedigree 1.E comprises the two brothers BUR212 and BUR258 and their unsampled parents. Both brothers carry the I2a1 chromosome Y haplogroup. Plague was detected in BUR212.

Pedigree 1.F

Generations represented: ?
Number of individuals (sampled): 3
Number of individuals (total): 3
Sampled males/females: 3/0 (100% males)

This pedigree consists of three males connected through unknown 2nd degree relationships. All carry the I2a1 chromosome Y haplogroup.

Pedigree 1.G

Generations represented: ?
Number of individuals (sampled): 2
Number of individuals (total): 2
Sampled males/females: 1/0 (100% males)

Pedigree 1.G is comprised by a male and an individual of unknown sex connected through an unknown 2nd degree relationship. The male carries the I2a1 chromosome Y haplogroup.

Pedigree 2.A

Generations represented: 5
Number of individuals (sampled): 11
Number of individuals (total): 17
Sampled males/females: 11/0 (100% males)

The largest pedigree of phase two, has a markedly different structure than the large

pedigrees of phase one. Pedigree 2.A is clearly patrilineal, consisting almost exclusively of fathers and sons. Interestingly, there is a persistent pattern in the pedigree, where two brothers (or half-brothers) are buried in the grave, but of each set of brothers, only one have their sons buried at the grave. All individuals in this pedigree carry the I2a1 chromosome Y haplogroup. Furthermore, the first generation of pedigree group 2.A is represented by two half-brothers (BUR307 and BUR276) who share the same mother, and whose fathers were related to each other. This is one of only two total examples of half-siblings in the dataset, which implies that having two reproductive partners was not the norm, or that the progeny from these unions were buried elsewhere, and thus also perhaps not socially accepted. Moreover, it is perfectly possible that this case of half-siblings represented misattributed paternity, and thus was not known in the community. This finding is relatively rare among comparable genetic studies, but not unheard of. While no half-brothers were found in the Neolithic site Gurgy from present day France23, half-brothers have been identified in Neolithic sites from the British Isles34 and Scandinavia15, and from later sites33,35.

Pedigree 2.B

Generations represented: 2

Number of individuals (sampled): 3

Number of individuals (total): 4

Sampled males/females: 3/0 (100% males)

Pedigree 2.B consists of an unknown father/son relationship between BUR323 and BUR235, and an unknown 2nd degree relationship between BUR323 and BUR326. The two individuals with sufficient chromosome Y coverage to call their haplogroup both carry the I2a1 haplogroup.

Pedigree 2.C

Generations represented: 2

Number of individuals (sampled): 2

Number of individuals (total): 4

Sampled males/females: 1/1 (50% males)

Pedigree 2.C comprises the two siblings BUR292 and BUR195 and their unsampled parents. BUR292 carries the I2a1 chromosome Y haplogroup.

Pedigree 2.D

Generations represented: ?

Number of individuals (sampled): 2

Number of individuals (total): 2

Sampled males/females: 2/0 (100% males)

Pedigree 2.D is comprised by two males connected through an unknown 2nd degree relationship. Both carries the I2a1 chromosome Y haplogroup, but have different mitochondrial haplogroups..

Pedigree 2.E

Generations represented: ?

Number of individuals (sampled): 3

Number of individuals (total): 3

Sampled males/females: 2/1 (66% males)

This pedigree consists of two males (BUR231 and BUR256) and one female (BUR306) connected through unknown 2nd degree relationships. Both males carry the I2a1 chromosome Y haplogroup, while BUR306 and BUR231 shares the same mitochondrial haplogroup.

Pedigree 2.F

Generations represented: 2

Number of individuals (sampled): 2

Number of individuals (total): 3

Sampled males/females: 1/1 (50% males)

This pedigree consists of a mother (BUR293) and her son (BUR230). The son has the I2a1 chromosome Y haplogroup.

Pedigree 2.G

Generations represented: 2

Number of individuals (sampled): 2

Number of individuals (total): 4

Sampled males/females: 0/2 (0% males)

Pedigree 2.G comprises the two sisters BUR318 and BUR196 and their unsampled parents.

Unrelated (phase 1)

Number of individuals: 15

Sampled males/females: 9/6 (60% males)

The unrelated individuals from phase one carry both the I2a1 and the H2a1 chromosome Y haplogroup. This group also has a slightly elevated frequency of females.

Unrelated (phase 2)

Number of individuals: 26

Sampled males/females: 17/9 (65% males)

The unrelated male individuals from phase two generally carry the I2a1 chromosome Y haplogroup, except for BUR282 who carries the G2a2 haplogroup.

Unrelated (unknown phase)

Number of individuals: 7

Sampled males/females: 5/2 (71% males)

The unrelated male individuals whose phase is uncertain all carry the I2a1 chromosome Y haplogroup

Supplementary Note 2.3 - Chromosome Y diversity

We investigated the diversity within the Y chromosome of all human samples analysed in this study by classifying haplogroups using the ISOGG database (see Seersholm et al. 2024, Supplementary Note 2 for details). We found that the majority of samples generated for this study belonged to either major haplogroup I and H, with a single individual belonging to haplogroup G (Extended Data Fig. 1a). In agreement with the results reported in the main paper, we find a strikingly different pattern between the two phases: While Phase 1 is diverse and dominated by three different haplogroups (H2a1, I2a1a1, and I2a1a2), Phase 2 is very homogenous, consisting almost exclusively of individuals with haplogroup I2a1a1.

To contextualise these results in a broader Western European perspective, we visualised the distribution of the two main haplogroups from Bury (I and H), together with haplogroup R associated with the spread of Steppe related ancestry across Europe (Extended Data Fig. 1b). From this plot, the rarity of haplogroup H and the westwards spread of haplogroup R after ~2,300 BC is very clear. The distribution of the three major subclades within haplogroup I form a less clear pattern. Generally, we find that all three subclades are widely distributed across Western Europe in both the time window of Phase 1 and in that of Phase 2. Furthermore, as indicated in the plot, there is no evidence for a single chromosome Y haplogroup associated with the northward spread of Iberian ancestry discussed in the main manuscript.

Supplementary Note 2.4 - Effective population size estimates

To assess if the populations at the two phases followed similar population size trajectories, we estimated effective population sizes projected back in time using the program IBDNe (v. 23Apr20.ae9)[95]. As input, we used IBD segments estimated with IBDseq[65] (see methods), from unrelated individuals stratified by phase. We ran IBDNe on this data using 12 threads and default parameters, and plotted the resulting population size estimates in R, converting generations ago (GEN) to absolute age using 28 years per generation and average ages of Phase 1 and 2 of 3250 and 2650 cal BC, respectively.

As depicted in Extended Data Fig. 4, the populations from each phase follow separate population size trajectories, strengthening our hypothesis of two largely distinct populations at Bury. Furthermore, while the uncertainties are large for the majority of the estimates, three main observations are well supported in the data: (1) the larger population size in phase 2 than phase 1 at the point of sampling, (2) the very recent decrease in population size for Phase 1 and (3) the generally more stable population in Phase 2. Yet, these results are biased by the different social organisation between the phases. Since Phase 1 consists of a single large family with few unrelated individuals, the effective size of this population would naturally be

very small. For Phase 2, the high number of unrelated individuals would drive the size estimate upwards.

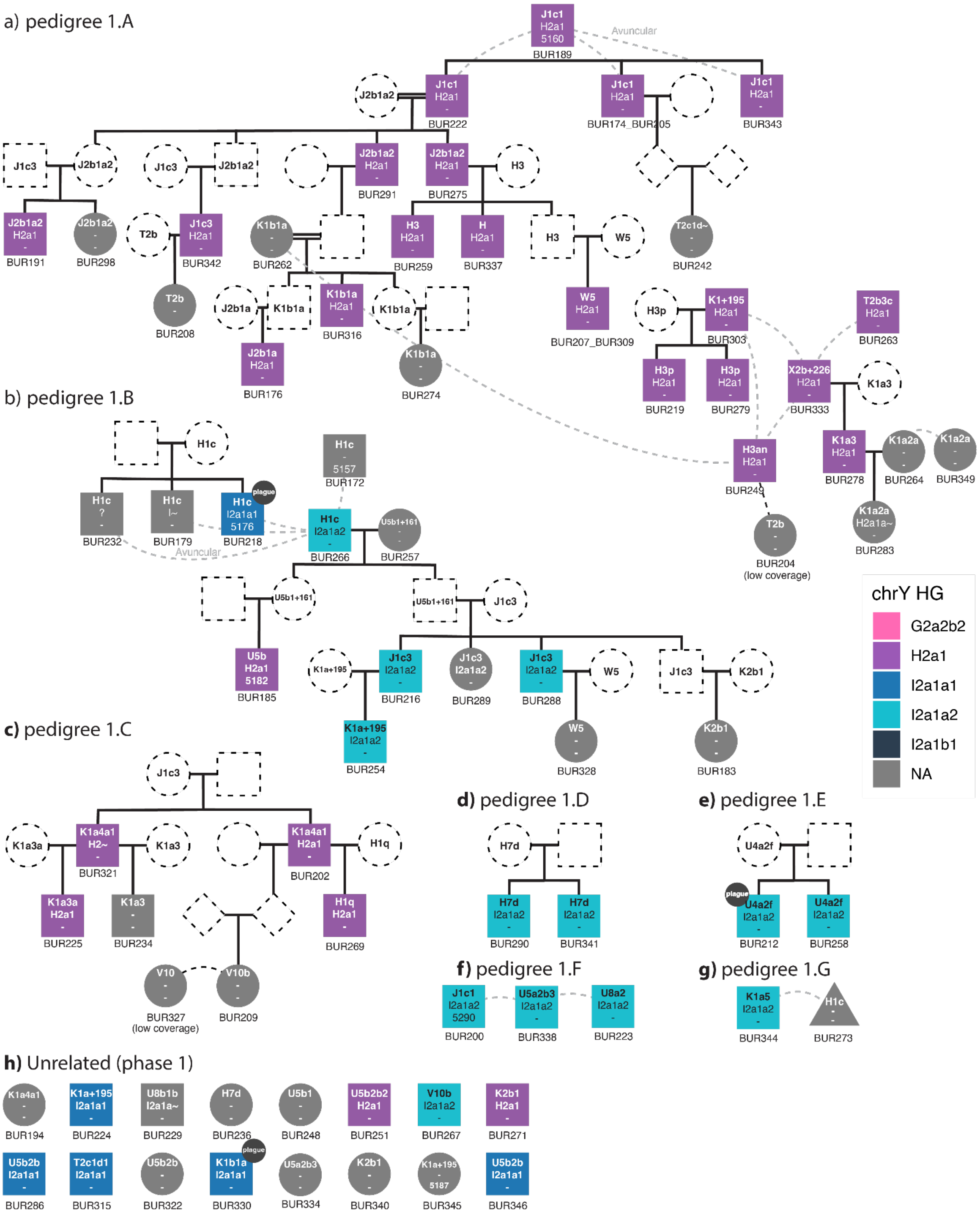

Supplementary Figure 2.1. Pedigrees from Phase 1 colored by Y haplogroup. Circles and squares represent females and males, respectively, while triangles represent unknown sex. Inside each shape, individuals are labelled with their mitochondrial haplogroup (first line), chromosome Y haplogroup (second line) and calibrated median age (last line). Colors represent chromosome Y haplogroup. Furthermore, solid black lines between shapes indicate well defined first degree relationships, while stippled black and grey lines specify unknown or uncertain first and second degree relationships, respectively.

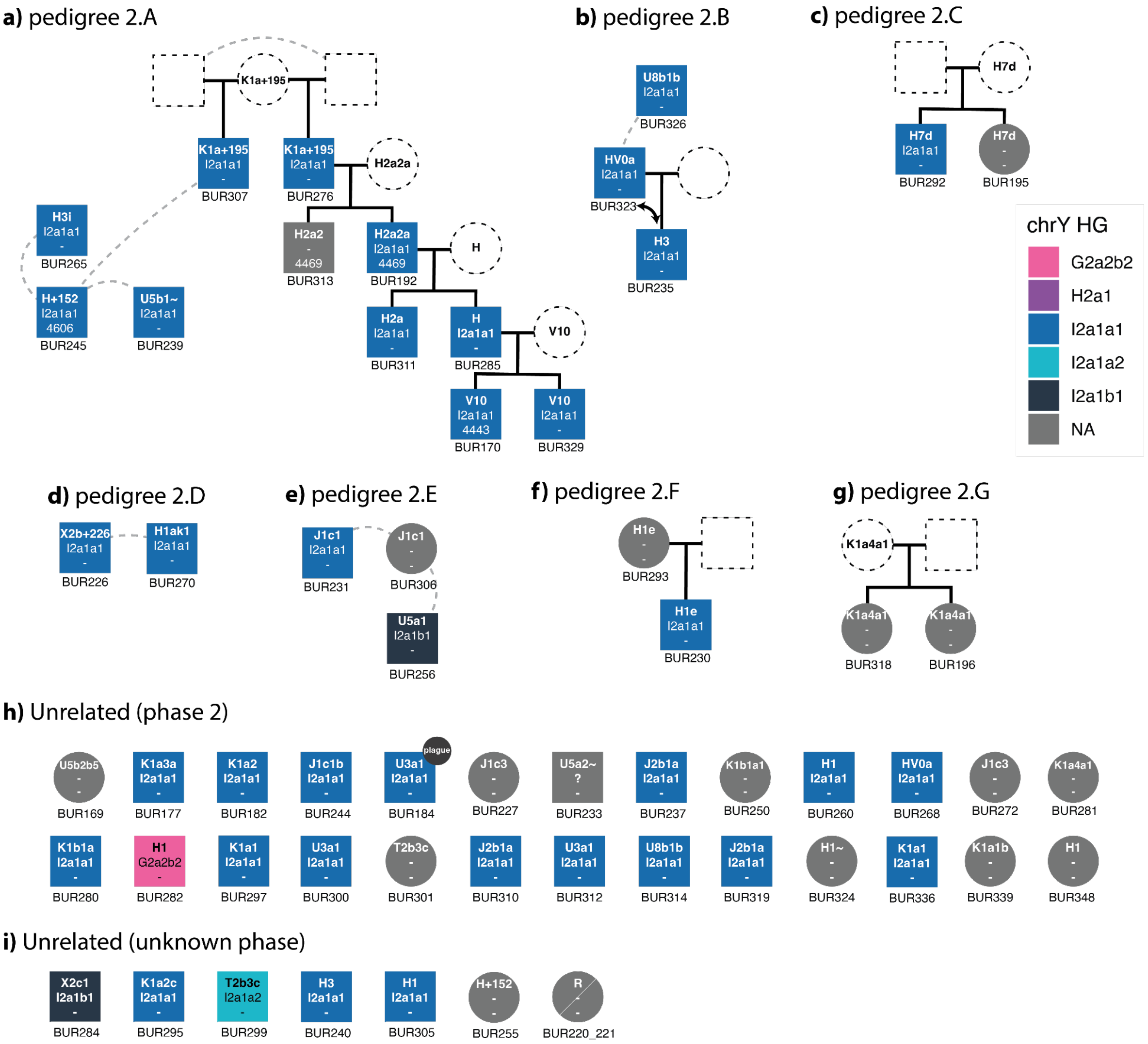

Supplementary Figure 2.2 Pedigrees from phase 2 colored by Y haplogroup. Circles and squares represent females and males, respectively, while colors represent chromosome Y haplogroup. Inside each shape, individuals are labelled with their mitochondrial haplogroup (first line), chromosome Y haplogroup (second line) and calibrated median age (last line). Furthermore, solid black lines between shapes indicate well defined first degree relationships, while stippled black and grey lines specify unknown or uncertain first and second degree relationships, respectively.

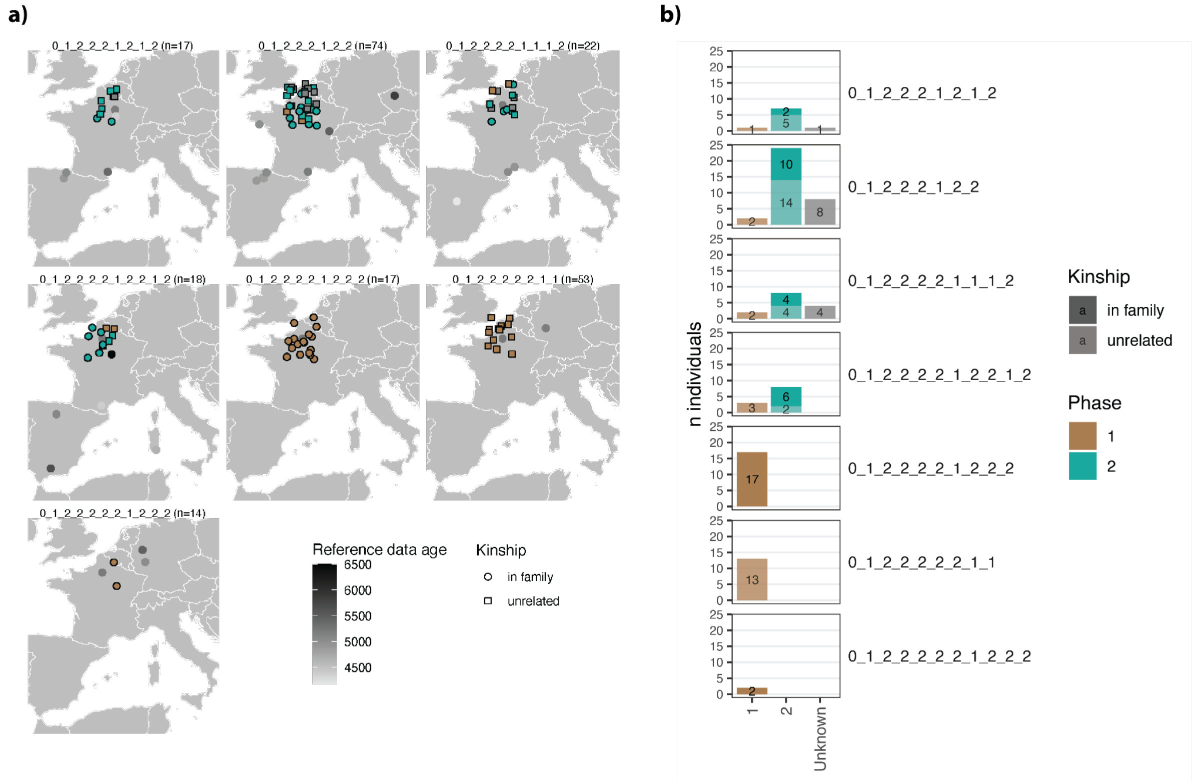

Supplementary Figure 2.3. IBD clustering results. a) Geographical spread and age of all samples within the seven clusters with any samples from Bury (using a cluster cut height of 4,000). Bury individuals are colored by phase (phase 1, phase 2 or unknown) while the reference data is coloured by age in shades of grey. The shape of the samples from bury indicates whether a given individual is unrelated (square) or part of a pedigree (circle). **b)** Stacked barplot of the number of unrelated/related samples from Bury within each cluster.

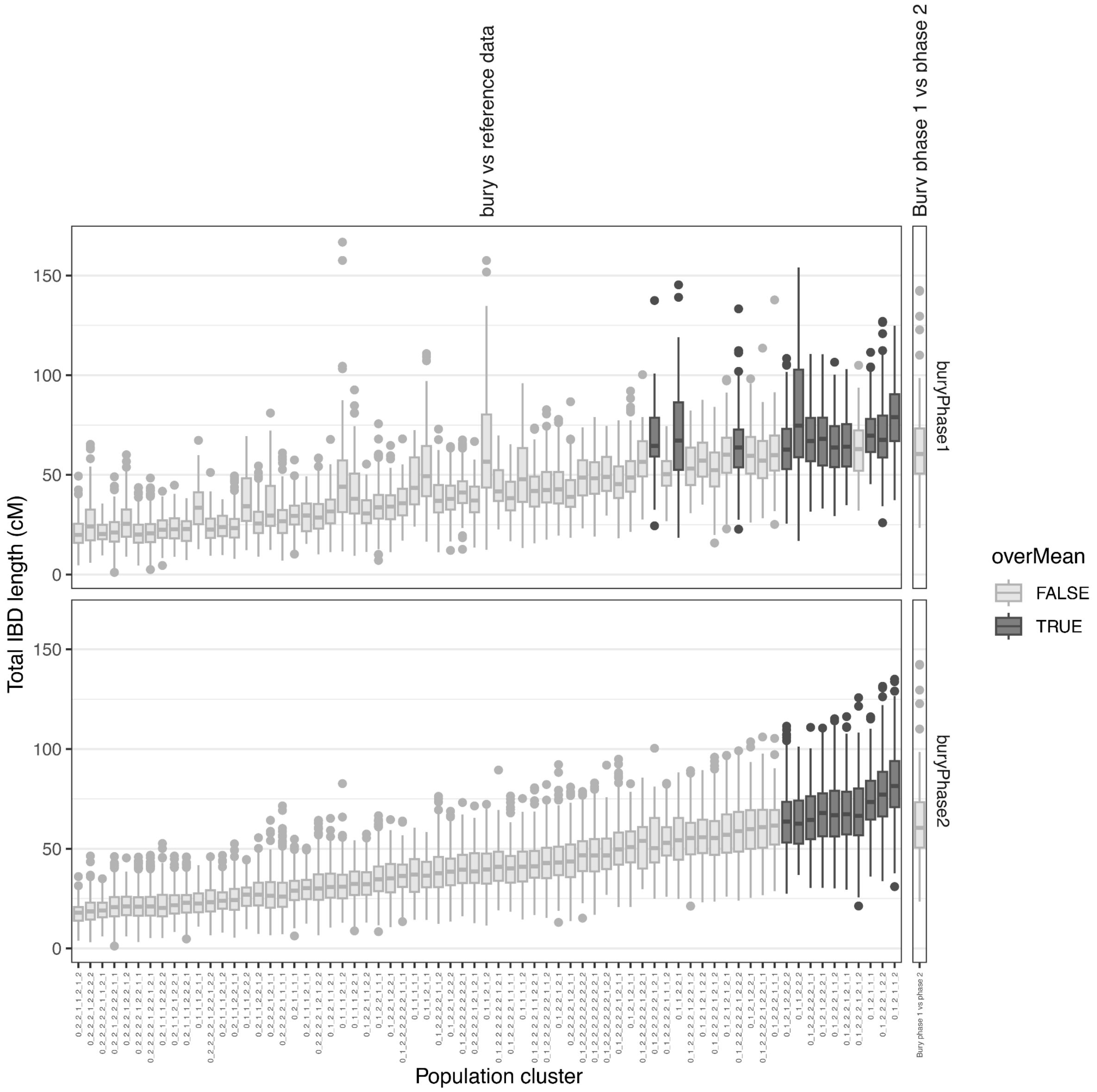

Supplementary Figure 2.4. IBD sharing between Bury phases and groups of Neolithic farmers. Top three 'global' clusters with highest IBD sharing against the Bury populations are shown. Each boxplot represents the IBD sharing between a 'minor' cluster and the populations of phase 1 or phase 2, respectively. Color indicates whether a given population has a higher mean IBD sharing than that shared between the two phases at bury.

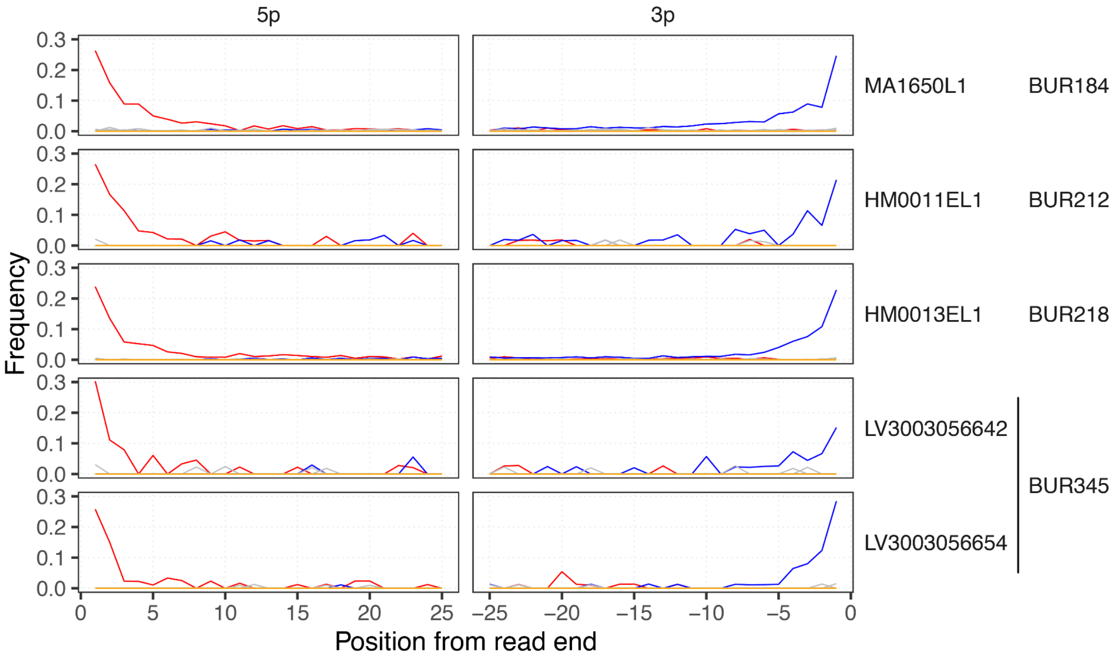

Supplementary Figure 2.5. Ancient DNA damage for *Yersinia pestis* reads. C>T and G>A misincorporations in the four plague positive individuals detected in this study. Only double stranded libraries that were not USER treated are shown.

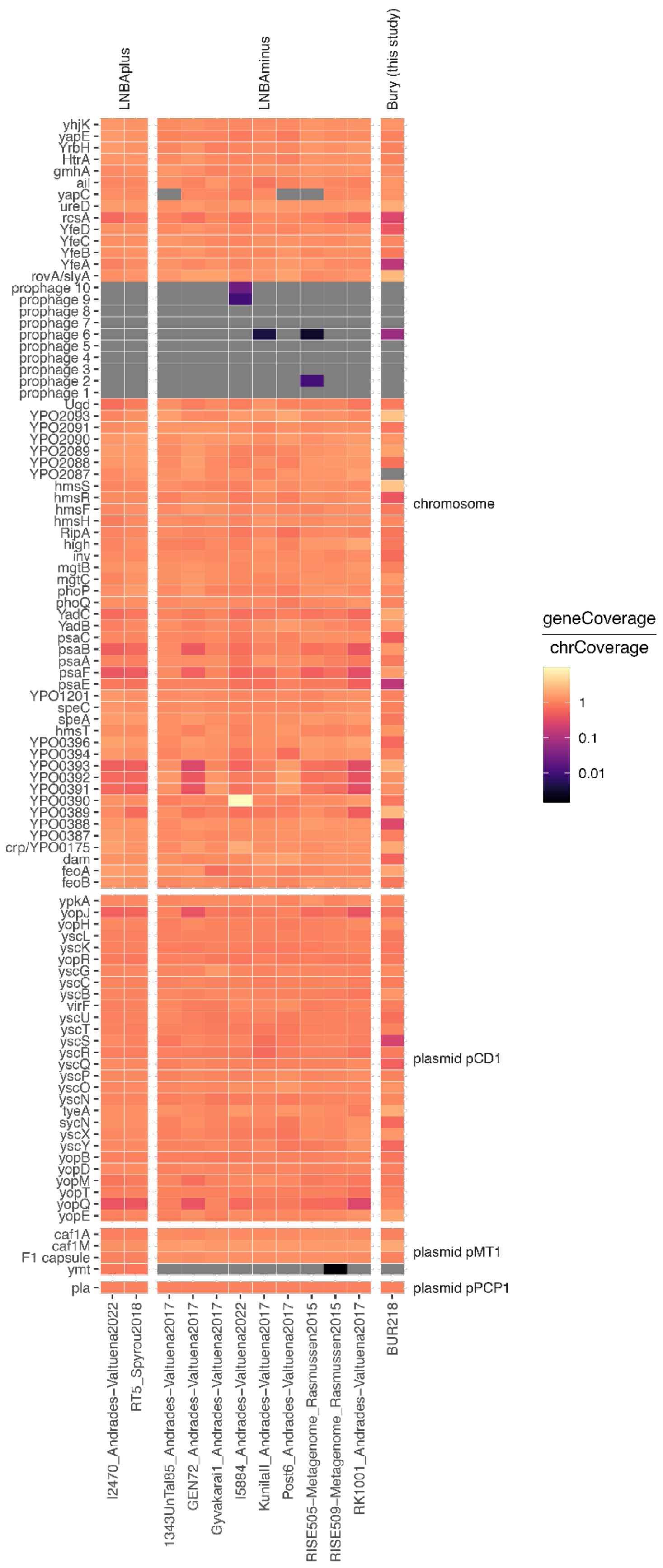

Supplementary Figure 2.6. Virulence gene coverage in BUR318. Color indicates per gene coverage normalised to the average plague coverage in the sample.

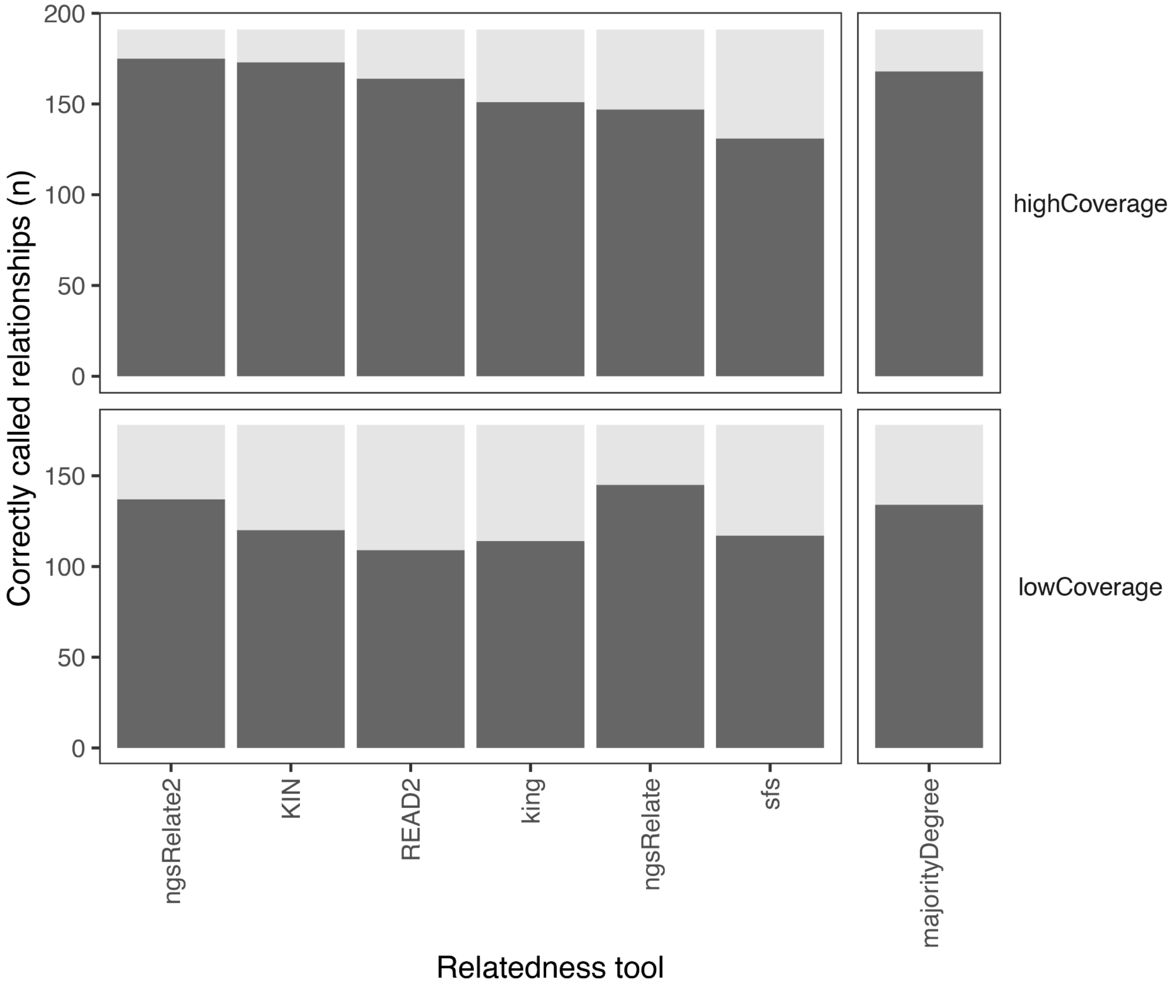

Supplementary Figure 2.7. Benchmarking relatedness estimation tools. 'highCoverage' represents relationships where both samples have over 0.1X coverage.

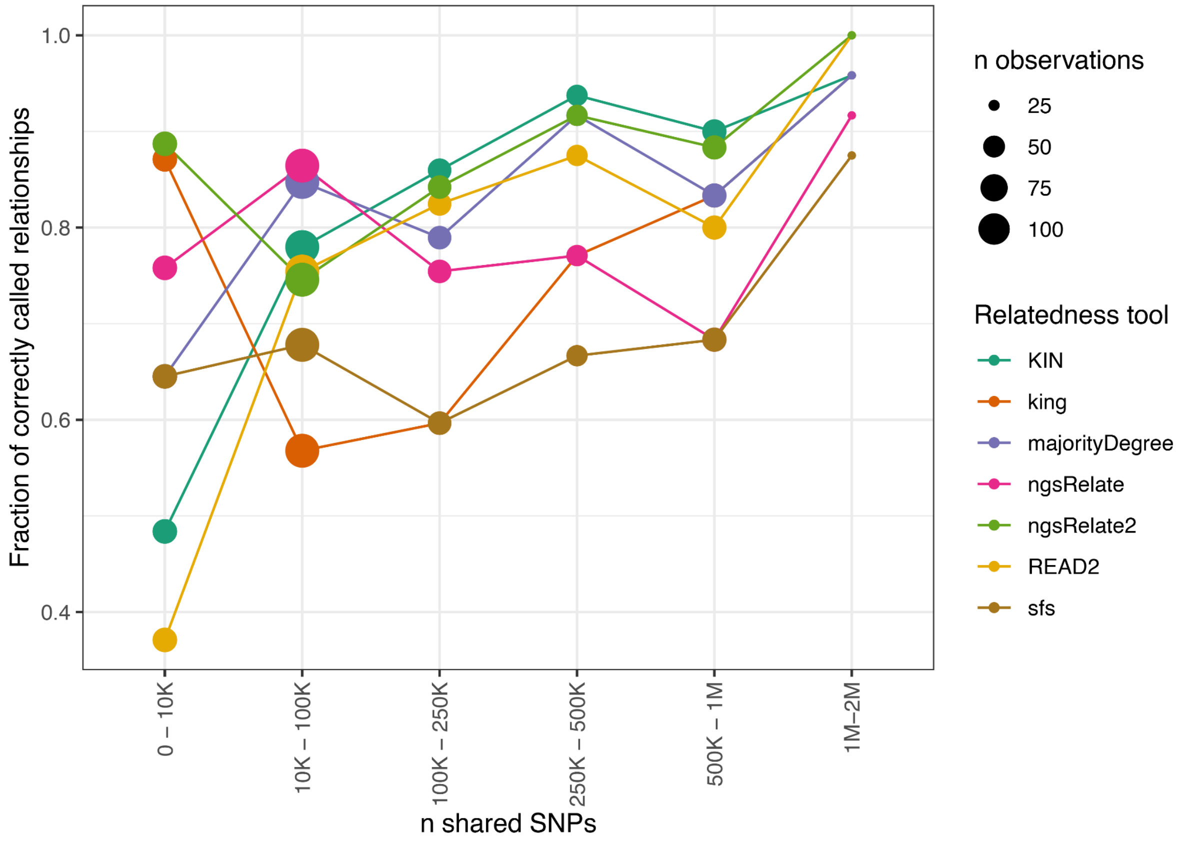

Supplementary Figure 2.8. Comparison of relatedness estimation tools, stratified by shared SNPs.

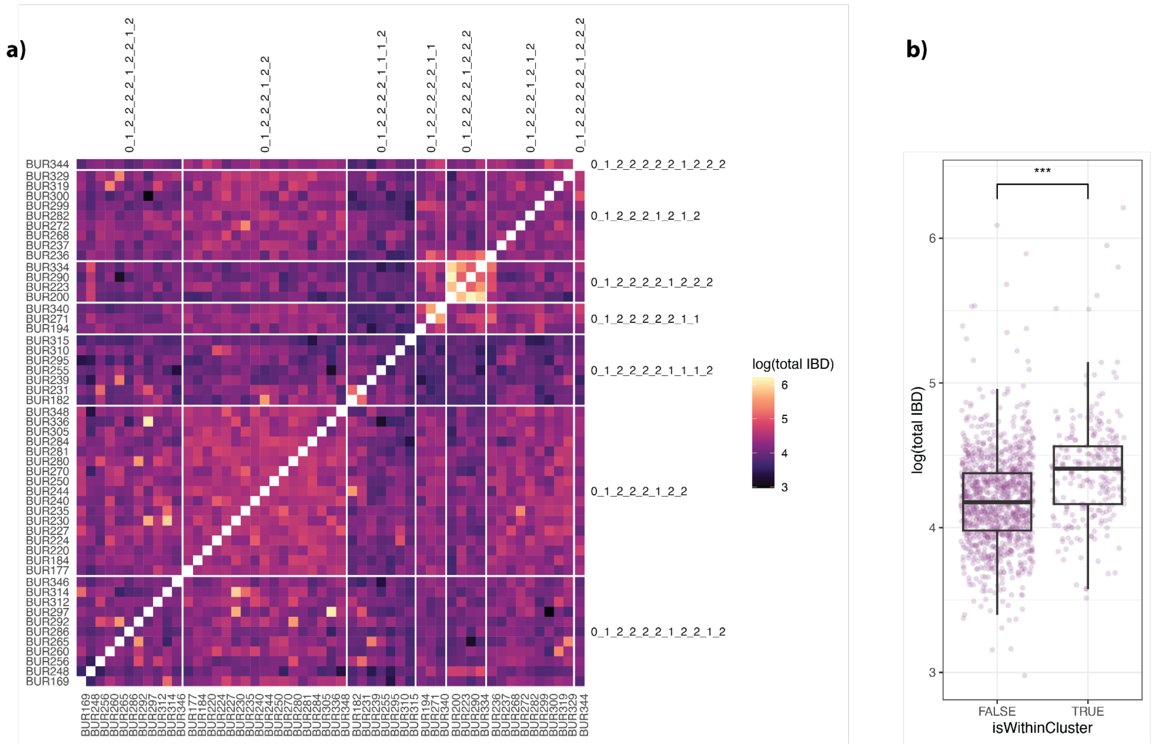

Supplementary Figure 2.9. Validation of the hierarchical clustering. a) Heatmap of shared IBD between all samples generated for this study stratified by genetic clusters. b) Violin plot comparing the amount of shared IBD within and between clusters (one-sided Wilcoxon rank-sum test: p-value < 2.2e-16, W=188,429 and effect size=0.09).

Supplementary Note 3 - Pollen results from the Paris Basin and Scania

Ralph Fyfe

Supplementary Note 3.1 - Vegetation history of the Paris Basin through the Neolithic (5300-2200 cal BC)

Reconstruction of vegetation history in Paris Basin draws mainly on floodplain sequences with some lake sequences which are largely produced by Leroyer, although little of this work is accessible through publications in the international literature, and the thesis of Leroyer (1997)[96] is not readily available online to explore the original diagrams. Synthesis chapters including summary pollen count diagrams have been published in French[97,98]. David (2014)[99] has drawn on the data of Leroyer (1997)[96] to produce quantified vegetation estimates using the REVEALS model[100] for western and northern France across the Holocene, with summary results from the Paris basin in David et al. (2012)[35]. As has been shown in other syntheses, the REVEALS results produce stronger evidence for human influence that the un-transformed pollen counts: the interpretation of Leroyer (2006)[97], based on pollen counts only, suggests only very limited localized transformations until the late Neolithic.

The REVEALS work of David et al. (2012)[35] for the Paris basin divides the Neolithic into seven time windows: 5100-4600 cal BC (the early Neolithic), 4600-4200 cal BC (middle Neolithic 1), 4200-3750 cal BC (middle Neolithic 2), 3750-3400 cal BC (middle Neolithic 3), 3400-2900 cal BC (late Neolithic), 2900-2500 cal BC (final Neolithic 1) and 2500-2200 cal BC (final Neolithic 2). It is necessary to aggregate pollen counts into time windows to generate regional cover estimates from multiple sequences. Both Leroyer (2006)[97] and David (2014)[99] are cautious to emphasize that, for the Paris basin, the influence of humans on vegetation is strongest from sites proximal to archaeological sites (cf. Mercuri et al. 2019)[101], or higher population densities, and that there were likely strong differences in human influence. Piecing together low resolution or fragmentary pollen records in regions such as this remains a challenge in synthetic work, and risks over-simplification of what are likely to have been heterogenous landscapes.

There is no clear evidence for transformations associated with the first Neolithic in the Paris basin (around c.5300 cal BC). Leroyer (2006)[97] states that whilst cereal cultivation is clearly evident in some of the pollen records, it is not accompanied by significant opening of forests. The influence of the first Neolithic farmers was not sufficiently strong to impact regional vegetation. The earliest Neolithic impact is visible in the REVEALS time window 5100-4600 cal BC, marked by increases in grassland (Poaceae) at the expense of woodland taxa (particularly reduction in *Corlyus*). The interpretation of David et al. (2012)[35] is the transformation of open hazel woodlands to fields and pastures starting in the 5100-4600 cal BC time window, and this is stronger in the REVEALS data than in the pollen counts[97]. From 4600-4200 (Middle Neolithic 1) there is stagnation in the regional cover of grasses and grassland indicators, suggesting a

continuity in land use. More detailed local interpretation of the pollen count data in Leroyer (2006)[97] describes strong variation in the influence of agropastoralists through the middle Neolithic, depending on the proximity of the pollen sites to known areas of activity (for example, localized clearance of alder to sustain grassland and settlement close to occupied areas).

In the Paris basin a clear transition occurs at 3750 cal BC, with clear reduction in tree cover and an increase in grassland, cereals and other ruderal taxa in the REVEALS results[35]. These indicators are sustained in the 3400-2900 cal BC time window, and are evidenced in both pollen count data and REVEALS results. Cover of grassland then drops significantly in the time window at 2900-2500 cal BC (halving from around 50% of the regional vegetation to 25% cover), and cereals and ruderal indicators also disappear. Agropastoral indicators return in the final Neolithic time window at 2500-2200 cal BC at similar levels to the middle Neolithic time windows, although overall openness remains low.

Supplementary note 3.2 - Summary regional vegetation cover from pollen diagrams from southernmost Scania

Data sources and methods

Four sites with detailed palynological records, and robust age-depth models based on radiocarbon dating, have been used to produce a quantified regional vegetation reconstruction for southernmost Scania. The sites are situated in the same area, close to the coast and in the proximity of known cultural material from the earlier Neolithic. The four pollen datasets have been extracted from Neotoma. The records from Bjärsjöholmssjön (medium-sized lake), Krageholmssjön (large lake) and Fårarps Mosse (a small bog) were generated as part of the Ystad project[102,103]. Bussjösjön (a small lake) lies between the other three sites that are used[104].

The pollen records were used to quantify regional vegetation abundance using the REVEALS model[100]. Each pollen dataset was harmonised using the protocols in Githumbi et al (2022)[105]. First, pollen identifications were harmonised to a set of 24 boreal and temperate European pollen morphological types for which relative pollen productivity estimates are available. These taxa typically represent more than 85% of the total pollen count from samples[106]. Second, samples were aggregated into eleven contiguous 200-year long time windows, between 4000 and 2000 cal BC (i.e. 4100-3900 cal BC, 3900-3700 cal BC etc). The REVEALS model was implemented using the REVEALS function within the LRA R package[107]. The function enables the use of deposition models for bogs (Prentice's model) and lakes (Sugita's model) and combines them to produce regional vegetation abundance estimates, and a gaussian plume model was used as the dispersal function, as the relative pollen productivity estimates were originally calculated using this method. The pollen morphological types were aggregated into six groups: primary forest, secondary forest, heath, cereals, other openland

and wetland.

Alongside the regional vegetation cover estimates, summed probability distributions (SPDs) of archaeological radiocarbon dates were generated as an independent proxy for human land use pressure. Only archaeological dates that have been classified to a particular culture were used. Separate SPDs were created for the TRB (342 dates from 93 sites), Pitted Ware (126 dates from 49 sites), Single grave (23 dates from 16 sites) and Scandinavian Late Neolithic (69 dates from 8 sites). Analysis was undertaken using the Rcarbon package[108].

Results and discussion

Supplementary Figure 3.1 shows the results of the REVEALS analysis for each 200-year time window, for the main relative pollen productivity taxa (i.e. those that comprise more than 1% of the regional vegetation cover), alongside the summed probability plots of the main cultures of the region. The regional vegetation at 4000 cal BC comprises a mix of primary forest (covering around 50% of the land area) composed of broad-leaved trees such as elm (*Ulmus*), lime (*Tilia*) and oak (*Quercus*), and light-demanding trees such as hazel (*Corylus avellana*) and ash (*Fraxinus excelsior*) that cover just over 40% of the land area. Open ground taxa most likely represent natural clearings in the forest and small patches of wetland. As the SPD for the TRB increases, primary forest is greatly decreased, down to 20% cover by 3700-3500 cal BC, with lime and elm almost totally absent. They are replaced by hazel (increasing to over 50% cover) and grasses (reaching 9% cover). Cereals are recorded in time windows 3900-3700 cal BC and 3700-3500 cal BC.

At time window 3300-3100 cal BC lime and elm recover, replacing hazel and reducing the amount of open ground, and by 3100-2900 cal BC lime covers reaches 20%. Open grassland at this time is reduced to less than 4% of the regional land cover, and the secondary forest type ash has increased. By 3100 cal BC the SPD indicates the presence of the Pitted Ware culture. Open grassland starts increases to over 10% regional cover from time window 2900-2700 cal BC, and marks an almost imperceptible decline in primary forest, replaced by increases in hazel. From 2700-2500 cal BC persistent cereal cultivation is recorded.

The results of the analysis demonstrate the close relationship between cultural development and change and land cover change in southernmost Scania, with the short-lived cultivation phase of the TRB aligned with reduced primary tree cover and the development of secondary woodland. Restructuring of woodland during the first Neolithic has previously been identified in southern Germany, promoting secondary woodland growth[109], and demographic changes have been associated with increasing landscape openness in Britain[110]. The disappearance of cereal from the pollen record cultivation in southernmost Scania, and re-establishment of primary woodland from the 3300-3100 cal BC time slice reflects natural successional processes over multiple centuries. Finer temporal scale analyses are needed to demonstrate

closer links between demographic changes and vegetation, along with information around subsistence practice from the archaeological record (e.g. Bevan et al 2017[111]), but the results support the assertion that changes in intensity of human activity control the nature of the regional vegetation.

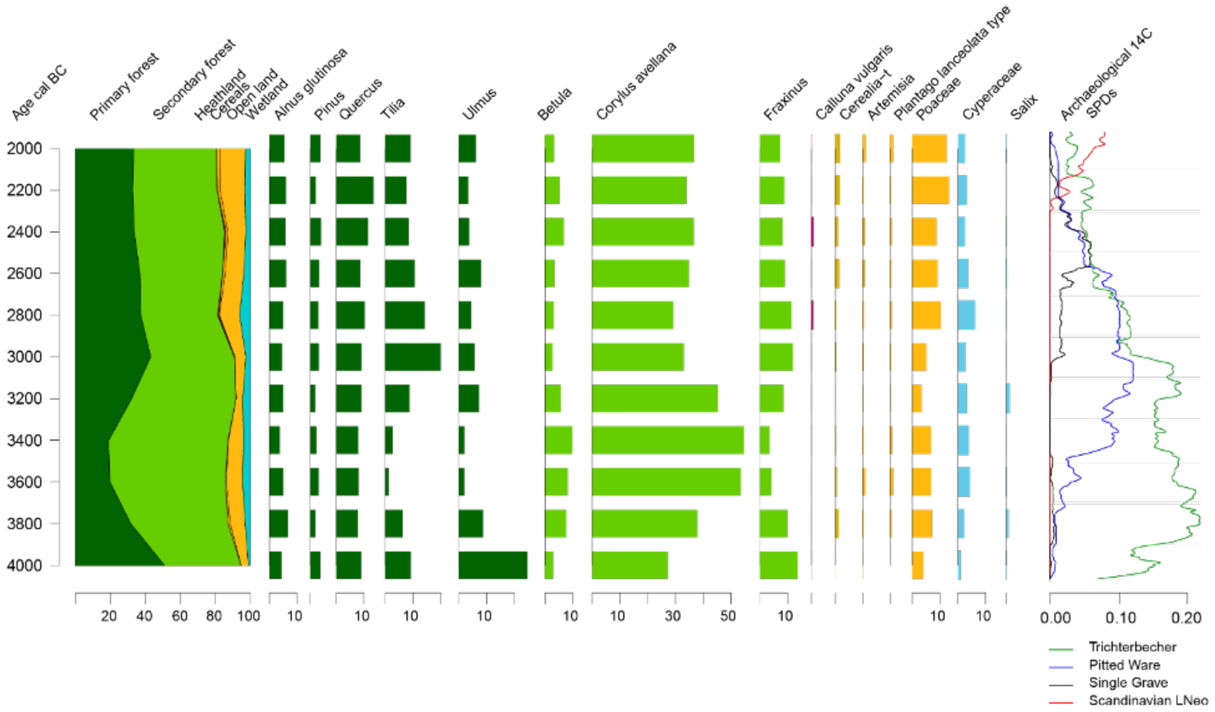

Supplementary Figure 3.1. Regional vegetation cover estimates for southernmost Scania from 4000-2000 cal BC, alongside summed probability density plots of the main cultures during this period. The summary plot includes primary forest (*Alnus glutinosa*, *Carpinus betulus*, *Fagus sylvatica*, *Juniperus*, *Picea*, *Pinus*, *Quercus*, *Tilia* and *Ulmus*), secondary forest (*Fraxinus*, *Betula* and *Corylus avellana* type), heath (Ericaceae and *Calluna vulgaris*), cereals (Cerealia-type (including *Hordeum* identifications) and *Secale cereale*), other openland (Chenopodiaceae, *Artemisia*, *Filipendula*, *Plantago lanceolata*, Poaceae, *Rumex acetosa* type) and wetland (Cyperaceae and *Salix*). The SPDs are based on: TRB 342 dates from 93 sites; Pitted Ware 126 dates from 49 sites; Single Grave 23 dates from 16 sites; and Scandinavian Late Neolithic 69 dates from 8 sites.

Supplementary Note 4 - Summary pollen diagrams from North Zealand

Morten Fischer Mortensen and Sascha Krüger

Methods

Four palynologically investigated and well-dated sites in Northern Zealand (Denmark) were chosen for quantitative landscape reconstruction. The four lakes (Kornerup, Dalby Sø, Højby Sø and Birkerød) are located in close geographical proximity and each features a similarly high resolution. For the individual data sets, REVEALS[100] was calculated using 20 pollen types. Most of them are assigned to one of the following groups for overview purposes: primary forest (*Alnus*, *Fraxinus*, *Picea*, *Pinus*, *Quercus*, *Tilia*, *Ulmus*), secondary forest (*Betula*, *Corylus*), open land (*Artemisia*, *Poaceae*, *Plantago lanceolata*, *Rumex*). Afterwards, a temporal binning of species was carried out in two hundred-year slices between 2000 and 4000 BC to allow for a sufficient quantity of samples per time slice.

The analyses were carried out using R and RStudio (vers. 4.2.2) with the packages *disqover* for calculating REVEALS in R[112] and *dplyr*[113] for the temporal binning of different pollen datasets.

Results and discussion

Supplementary Figure 4.1 displays a summary of the contribution of the respective species to the vegetation cover in two-hundred-year slices. For improved readability, the figure is divided into cultural-historical periods and the time range between 3000 and 2800 BC is colour highlighted.

Between 4000 and 3000 BC, a gradual trend emerges. Species of the primary forest on nutrient-rich soils, such as *Tilia* and *Ulmus*, are considerably reduced and pushed to niches by 3200 BC. They are replaced by open-land species, which are classically categorised as farming indicators (Cereals) and pastoral indicators (*Plantago lanceolata*, *Rumex*)[114]. Hazel likewise benefits from afforestation. The above-mentioned human indicators register an increasing trend up to 3000 BC, with the highest values for cereal pollen so far.
In the time slice 3000 to 2800 BC, a drastic discontinuity of the previous trend of vegetation development can be observed. All species of the primary forest (except for *Alnus*) increase by more than double in some cases. At the same time, secondary forest values decrease and cereal pollen is almost completely absent from all records. Pastoral indicators, too, decline sharply but remain represented.

Between 2800 and 2000 BC all human indicators rise again and exceed the levels before 3000 BC. Evidence of cereal cultivation resumes abruptly, although the peak in primary forest

density is not exceeded until 2600 BC. Until 2000 BC, the trend of landscape opening combined with a predominant reduction of primary forest continues.

The Neolithic landscape opening in northern Germany and Denmark is closely bound to FBK[36,115–121] Rasmussen, 2005; Rasmussen and Bradshaw, 2005). This is also reflected in Supplementary Figure 4.1 between 4000 and 3000 BC. Trees on nutrient-rich soils give way to the cultivation of cereals, timber production and the creation of pastoral land. However, in the time slice 3000 to 2800 BC this trend is reversed. The absence of cereal pollen and the significant increases in *Tilia*, *Ulmus* and *Quercus* suggest that the primary forest reclaimed areas previously used for agricultural purposes. The secondary forest is likewise declining, indicating an overall densification of the near-natural forests. Pastoral indicators are still present, however, they are equally part of the natural vegetation and do not necessarily indicate livestock presence. The time slice between 3000 and 2800 BC thus describes a significant decline in human activity in Northern Zealand.

The peak in the spread of primary forest in the following zone (2800-2600 BC) can probably be explained by a calculation effect caused by the time slice boundaries. In this phase, clear signs of human activity appear contemporaneously.

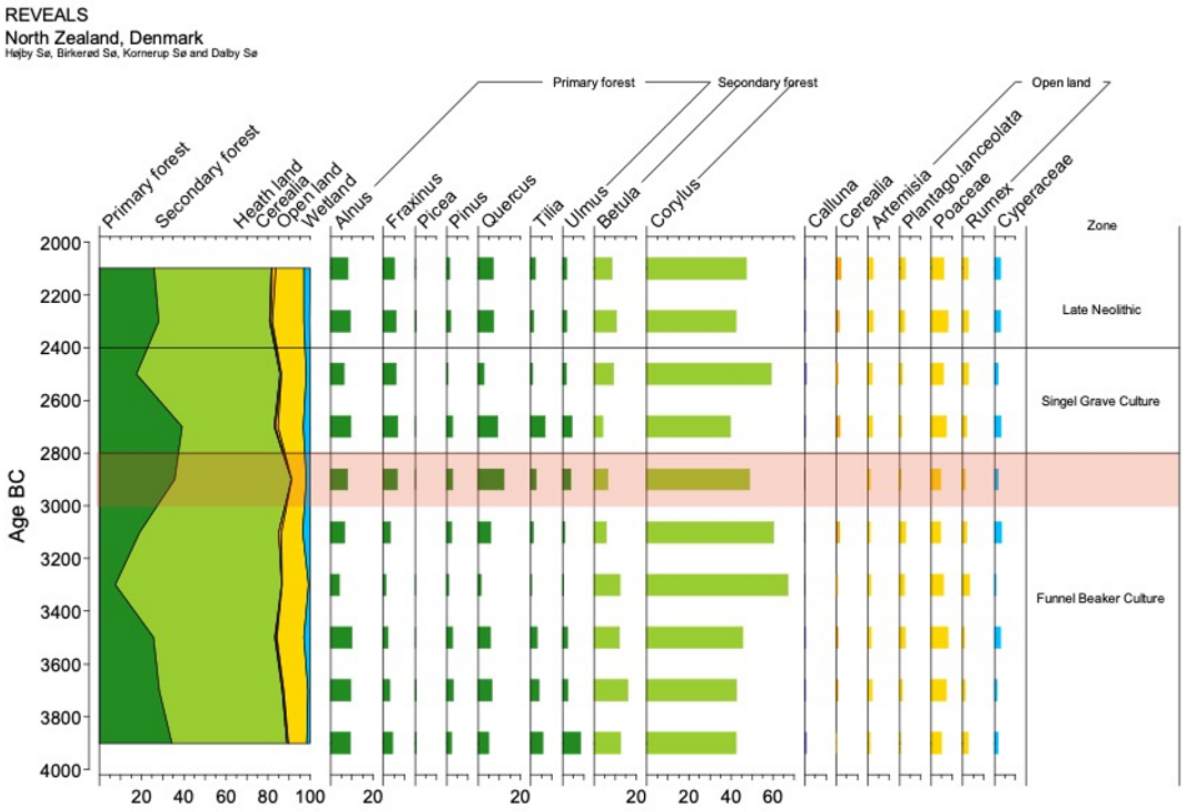

Supplementary Figure 4.1. Summary of the contribution of the respective species (divided into primary forest, secondary forest, and open land) to the vegetation cover in two-hundred-year slices. Cultural historical periods are shown on the right, and the time range between 3000 and 2800 BC is colour highlighted.

Supplementary Note 5 - The relationship between the megalithic graves of the Funnel Beaker Culture and the subsequent Late Neolithic stone graves

Torben Dehn and Svend Illum Hansen

For many decades, researchers have speculated about the sudden cessation of megalith construction, after a relatively brief period of intensive building activity. Among the subsequent grave forms that appear, stone constructions continue. However, they have a wholly different character. This shift is demonstrated in the following account, based on thorough knowledge of megalith construction, achieved through nearly 40 years of reconstruction and analysis, whereas observations about Late Neolithic (LN) stone cists are primarily based on descriptions in the literature[122–126].

In Denmark the Funnel Beaker Culture's megaliths are built according to relatively restricted principles, which are actualised in varied monument types, with the use of different materials and technical solutions. It is clear that some stone graves – even within the same area – are built with greater care than others. Yet, all share the same intention, tradition and thought-process. Concisely expressed, the burial chamber must be completely sealed and dry. This has been achieved through distinctly sophisticated construction solutions through the entire span of the Funnel Beaker Culture[127]. The well-developed knowledge underlying the construction of megalithic graves is transmitted successfully over many generations, but it disappears when megalith construction ceases. When a stone-building tradition re-emerges several hundred years later, it is at a less sophisticated level of complexity.

The following LN stone grave tradition – known as burial cists, stone cists or slab cists – includes a much more heterogeneous group, in terms of construction, than the Funnel Beaker Culture stone grave tradition. There are greater regional differences, and it is difficult to identify focused construction principles, beyond that of building cists from flat stones. The stone graves from this period occur simultaneously with other grave constructions, including wooden coffins. Moreover, because stone cists were also built and used in the Bronze and Iron Age, with many LN structures re-used through later periods, their dating may often be unclear. A noticeable difference between the Funnel Beaker Culture and LN stone graves is that, the former involved raising primary, stand-alone monuments, whereas the latter were often secondary constructions in existing mounds.

Yet, a subtle relevant difference between stone graves in the two periods is that the FBC megalithic graves are always placed on existing surfaces, and while exceptions occur, by far the largest proportion of LN cists are either placed in virgin terrain or previously built mounds.

In comparison to the megalithic graves, which exhibit uniform technical solutions across time and space, the LN stone graves are based on regional traditions that differ in construction strategies.

FBC dolmens and passage graves are typically built as primary installations in newly built mounds. While LN cists can also involve entirely primary construction, the majority are secondary additions to existing features, often with several being added at a time. Concerning the FBC megalithic chambers, the surrounding mound offers maximal stability and sealing effect. This is not the case for LN cists. Most of them are placed secondarily in pits, where the cists are sealed with smaller stones, surrounded by additional supporting stones or stone-packings. In cases where the cists are a primary grave construction in a mound, the mound's turf layer is only stacked around the cist itself. This has been illustrated for the stone cist at Blære, which is the most recently excavated and best preserved case (Fabech 1988, p. 49, Fig. 5). That is to say that, around the cist – but not in the mound – measures are taken to prevent water seepage, and with it, erosion of the grave feature. In the mounds built with megalithic structures, in contrast, a series of components immediately around the stone structure are typically chosen, including roof shingles, flint-packings and inclined clay layers [128]. These measures are taken to prevent deterioration of the grave monument. An additional detail is that megalithic graves typically have dry-wall constructions, with dressed stone shingles between the wall-stones. Similar dry-wall construction occurs very rarely in LN stone cists, and when it does, only unmodified fieldstones are used.

In all circumstances, the megalithic grave's construction principles and building methods must be considered as both highly sophisticated and well defined, markedly different from those of LN cists, which themselves comprise a much more internally variable category. As mentioned above, the FBC megalithic grave's construction as a primary installation, with a stone chamber or dolmen placed in its own surrounding, newly built mound. In contrast, the LN cist's installation is relatively rarely a primary feature. Rather, it is most often placed in an existing structure, including in megalith-mounds. The LN cist's construction is, overall, simpler and not nearly as labour-intensive as that of the FBC megalithic graves. It does not appear to be the case that LN cists were built as part of such an integrated and constrained tradition as that of megalithic grave-construction, where a clear development from the earliest dolmens to passage graves may be seen. The construction of the simplest type – LN cists – appears to be more improvised. According to stone-grave building techniques, it appears that a fundamentally different way of thinking replaces the earlier one.

In conclusion, it may be asserted that the complicated technological and architectural praxis behind megalithic graves ceased to be transmitted, disappearing suddenly around 3,000 BC. The latest documented megalith raised in Denmark is dated to ca. 2950 BC, consistent overall with ^{14}C data from other parts of southern Scandinavia. Below we summarise the Danish

dates, along with two from Scania, Sweden, based on birch bark inserted in dry-wall under the construction[129].

Site	Lab no.	BP uncal	1 sigma	BC cal from	BC cal to
Hvalshøje	Ka-6976	4620	55	-3617	-3105
Snibhøj	AAR-5473	4590	40	-3514	-3104
Rævehøj	Ka-7000	4540	45	-3484	-3095
Jordhøj	K-978	4490	120	-3516	-2898
Jordhøj	Ka-7001	4485	50	-3361	-3016
Ubby Dysselod	Ka-6978	4475	45	-3356	-3015
Maglehøj	Ka-6975	4440	50	-3336	-2925
Ljunghög	LuS-12990	4435	50	-3335	-2922
Øm	AAR-8723	4420	40	-3330	-2916
Örenäs	X9-R	4415	55	-3333	-2911
Olshøj	AAR-7975	4315	60	-3307	-2702
Olshøj	AAR-5472	4245	40	-2923	-2675

Supplementary Table 5.1. Birch bark dates from primary construction of passage graves

Supplementary Note 6 - Isotopic proveniencing of human remains

T. Douglas Price

Supplementary Note 6.1 - Principles and Procedures

Strontium

Strontium isotope analysis provides a robust means for examining past mobility. Strontium moves into humans from rocks and sediment through the food chain[130–132] and deposited in the skeleton. The enamel in teeth forms in early childhood and contains the strontium isotope ratio of the food consumed and the local geology from the first years of life[133]. The ratio in the enamel remains largely unchanged during life and after death. The enamel is used as a signal of place of birth. If an individual moves to a new location in a different geologic context, or is buried in a new place, the enamel isotope ratio will differ from the new location, allowing the designation of that individual as non-locally born[134].

The strontium isotope ratio of $^{87}Sr/^{86}Sr$ varies among different kinds of rocks, based on their age and composition. The heavier isotope (^{87}Sr) is formed by the radioactive decay of rubidium-87. Thus, older rocks and sediments with more rubidium have higher $^{87}Sr/^{86}Sr$ values, while younger materials with less rubidium are at the opposite end of the range with lower ratios (e.g., Faure and Mensing 2004[135]). The proportion of ^{87}S varies in the terrestrial ecosystem, but averages around 7% of total strontium; ^{86}Sr is about 10%. Their ratio normally varies from about 0.700 in rocks with low Rb to 0.730 and much higher in high-Rb rocks that are billions of years old. Most measurements of human enamel fall in the range of 0.705 to 0.730.

Strontium isotope analysis for information on prehistoric residential mobility requires samples of dental enamel. Enamel powder for isotopic analysis is collected from the teeth by first burring the area to be sampled to remove possible surface contamination and then extracting a cusp or fragment in the case of friable enamel.

Any remaining dentine is carefully removed and the sample is ground to powder. The powder is weighed and submitted for measurement.

Measurement of $^{87}Sr/^{86}Sr$ in the enamel powder is done in the Geochronology and Isotope Geochemistry Laboratory (Dept. of Geological Sciences, University of North Carolina-Chapel Hill). Samples are dissolved in nitric acid and the strontium fraction purified by ion selective chromatography (Eichrom Sr resin) prior to analysis by TIMS on a VG Sector 54 mass spectrometer run in dynamic mode. Internal precision in the laboratory is consistently around 0.0007% standard error (or $1\sigma=0.00006$ in the ratio of a particular sample). Long-term,

repeated measurements of SRM-987 are around 0.710260—an acceptable difference from the recognized value of 0.710250—and raw sample values from individual runs are standardised to the recognized value of SRM-987.

Local Baseline

An essential aspect of strontium isotope analysis involves the determination of the local strontium isotope level[136,137]. The actual level of strontium isotopes in human tissue may vary from local geology for various reasons. It is necessary to measure *bioavailable* levels of $^{87}Sr/^{86}Sr$ to determine regional and local strontium isotope ratios for comparison with the human remains. Such measurements are used as a baseline for comparison with tooth enamel.

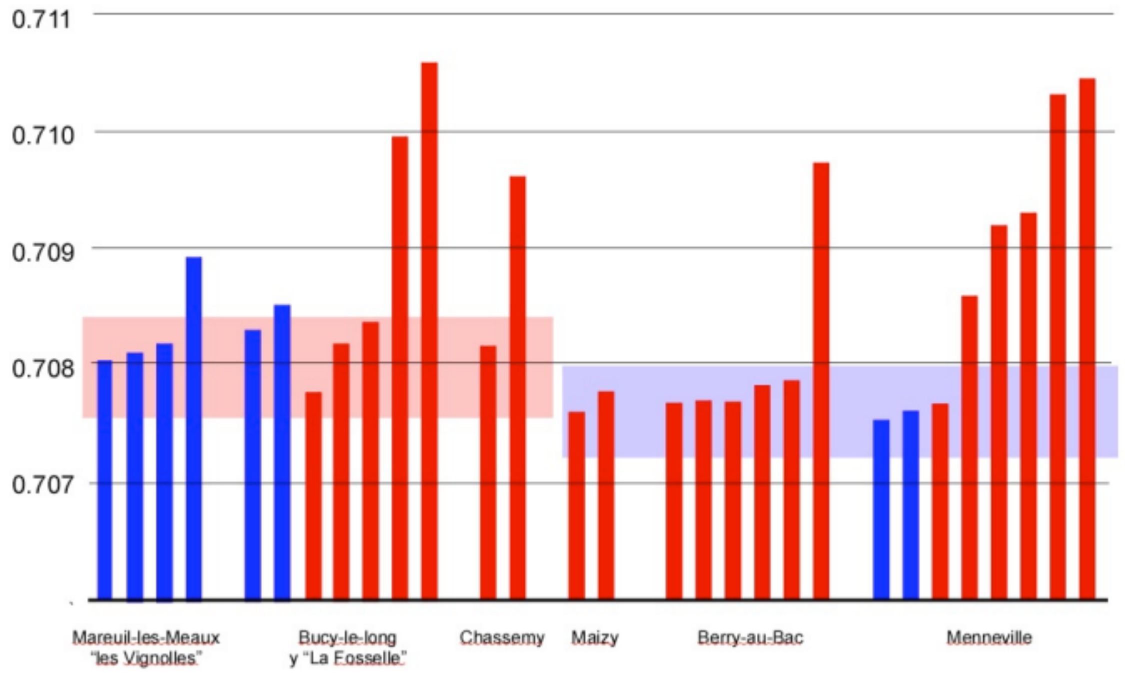

Supplementary Figure 6.1. Bar graph of ranked $^{87}Sr/^{86}Sr$ in human tooth enamel and fauna (blue) from Rubané (LBK) Early Neolithic sites in the Aisne Valley.

Bury is located 50 km north of Paris on a small tributary of the Oise River. The Paris Basin as a whole is characterised by $^{87}Sr/^{86}Sr$ values between 0.708 and 0.710, which fit well with the geology of the landscape and the deposits of loess that occur intermittently across the area (Fig. 1). Loess deposits are widespread in northern France and locally very thick (up to 12 m). The primary accumulations of typical calcareous loess date from the Upper Weichselian and Upper Saalian stages of the Pleistocene[138]. The deposits of LGM European loess in this area likely average 0.709-0.710[139] and would be a strontium source for human diets. These values fit well with our studies in the Aisne and Marne Valleys, also in the northern Paris Basin. We have measured $^{87}Sr/^{86}Sr$ in human tooth enamel from Rubané (LBK) Early Neolithic sites in the Paris Basin. The Aisne River is a left tributary of the Oise. A bar graph of ranked strontium

isotope ratios from these sites is shown in Supplementary Figure 6.1. Baseline values in this area ranged between 0.7072 and 0.7085.

Bury sits near the boundary between Tertiary and Cretaceous marine deposits in the Paris Basin (see Figure 2.5a in Maréchal & Rouillard 2020[140]). Comparison with the strontium isotope ratio for marine deposits from these periods[141–143] provides a range from 0.707 to 0.709 (see Figure 8 in Willmes et al. 2018[144]). Modern atmospheric dust in this area falls between 0.709 and 0.714[145] but is unlikely to contribute to prehistoric values. The IRHUM (Isotopic Reconstruction of Human Migration) project has begun the development of a database for bioavailable strontium isotope ratios for France[146,147]. This study lists values from 0.707 to 0.717 for the Paris Basin Supplementary Figure 6.1). Three of the sampling sites for the IRHUM project for France are located near the town of Bury (3, 6, and 10 km distant) and produced $^{87}Sr/^{86}Sr$ values of 0.7077, 0.7091, and 0.7094. In reality, the local baseline for most of northwest France probably ranges from 0.7075 to 0.710. The Wilmes et al. (2018[144]: Fig. 8) map of bioavailable $^{87}Sr/^{86}Sr$ indicates values between 0.7075 and 0.7103 in the area of Bury, based on soil leachates and plants (see Figure 8 in Willmes et al. 2018[144]).

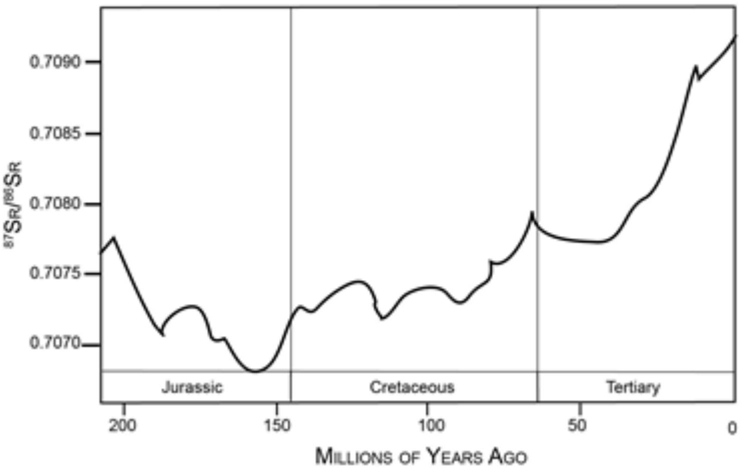

Supplementary Figure 6.2. Strontium isotope ratios in seawater over geological time (after Jones & Jenkyns 2001[141], Fig. 1).

Oxygen

Oxygen has three isotopes, ^{16}O (99.762%), ^{17}O (0.038%), and ^{18}O (0.2%), all of which are stable and non-radiogenic. Oxygen isotopes are much lighter and have a much greater relative mass difference than strontium isotopes (^{18}O is 12% heavier than ^{16}O; ^{87}Sr is 1% heavier than ^{86}Sr), making them, in sharp contrast to strontium, highly sensitive to environmental and biological processes. Oxygen isotopes, which are commonly reported as the per mil difference in $^{18}O/^{16}O$ between a sample and a reference material, can be measured in either the carbonate (CO^{-2}) or phosphate (PO^{-3}) ions of bioapatite. This value is designated as 618O. Two different

reference materials have been used, one for water (Vienna Standard Mean Ocean Water [VSMOW]) and one for solids (VPDB). These values can be equilibrated using a formula from Chenery et al. (2012)[148].

In bones and teeth, isotope variation in 618O due to physiological factors (e.g., perspiration, metabolic rate, and urine) is averaged, with variation among local populations generally less than 2‰[149]. Oxygen isotopes in the skeleton reflect that of body water[150,151], which in turn predominantly reflects that of local rainfall. Isotopes in rainfall are greatly affected by enrichment or depletion of the heavy ^{18}O isotope relative to ^{16}O in water due to evaporation and precipitation. Major factors affecting rainfall oxygen isotope ratios are latitude, elevation, and distance from the evaporation source (e.g., an ocean)—that is, geographic factors. Lachniet and Patterson (2009)[152] analysed 618O in surface waters collected from Guatemala and Belize. Their data show that temporally there is also an inverse correlation between rainfall amount and 618O. Spatially, two variables—distance from the coast and mean catchment altitude—explain 84% of the surface water 618O variability.

Like strontium, oxygen is incorporated into dental enamel—into both carbonate and phosphate ions—during the early life of an individual, where it remains unchanged through adulthood. Oxygen isotopes are also present in bone apatite and are exchanged through the life of the individual by bone turnover, thus reflecting place of residence in the later years of life. Thus, oxygen isotopes, although nonradiogenic, have the potential to be used like strontium to investigate human mobility and provenience. Oxygen isotopes have been employed in a number of studies[149,153–158].

Supplementary Note 6.2 - Le Tumulus des Sables

To better contextualise the findings at Bury, we decided to include data from the site Le Tumulus des Sables here. The burial mound of Le Tumulus des Sables, near Bordeaux in southwest France, dates from the Neolithic to the Iron Age, and is one of the few Neolithic sites from present-day France where extensive Strontium work has been conducted. Isotopic analyses of human teeth have been used to investigate the diet and mobility of the occupants[159]. 613C and 615N values (from dentine collagen) suggest a predominantly terrestrial diet for the population, unchanging through time. This suggests the diet of the inhabitants remained terrestrial throughout the lifetime of the site, despite its close proximity to the Gironde Estuary and the Atlantic Ocean and suggests limited mobility.

Twenty-five teeth (18 permanent, 7 deciduous) from Le Tumulus des Sables were analysed for Sr isotopes (both enamel and dentine). Fifteen of these teeth (14 permanent, 1 deciduous) were analysed for 618O. $^{87}Sr/^{86}Sr$ (on enamel and dentine) and 618O (on enamel) values are consistent with occupation of the surrounding region, with one individual having a 618O value consistent with a childhood spent elsewhere, in a colder climate region. The results showcase the complex reuse of this burial mound by a mostly local population over a period of about

2000 years.

The isotopic data suggest that the majority of individuals came from the local region, with only one individual identified as a non-local. Similar results have been found at other Neolithic sites in France and at Bell Beaker sites in the United Kingdom.

Supplementary Note 6.3 - Results of Analysis

Information on the samples we selected and the isotope ratios measured is provided in Supplementary Table 6. The average value for the 180 samples we have measured ± 1 s.d. was 0.7091 ± 0.0009, with a minimum of 0.7078 and a maximum value of 0.7181. The range of values for most of the samples was not great and it is likely that these could be found in the general range of values from northwestern France. Recall that the local baseline for northwest France is approximately 0.7075 to 0.710.

At the same time, it is clear that there are isotopic differences among the individuals buried at Bury. Fig. S5.5 is a bar graph of ranked $^{87}Sr/^{86}Sr$ values for 180 samples of human tooth enamel from the collective tomb at Bury. If we use breaks in the curve of values in Fig. S5.5 there are three values at the low end of the range and 11 values at the high end. The three low values come from burials BUR186 (Ind. 46), BUR294 (E12-203), BUR257 (G5-3159) and BUR296 (G5-2136), all below 0.7082. There are 11 samples from the high end that include values greater than 0.71024. These burials include BUR314 (F6-117), BUR335 (H27-308), BUR315 (H27-74), BUR203 (F9-1100), BUR324 (G11-38), BUR346 (H4-72), BUR312 (G6-1988), BUR213 (F 10-1203), BUR244 (E13-646), BUR323 (F5-423), and BUR295 (G6-2902). It is clear that the value of 0.7181 makes the last burial (G6-2902) quite distinct.

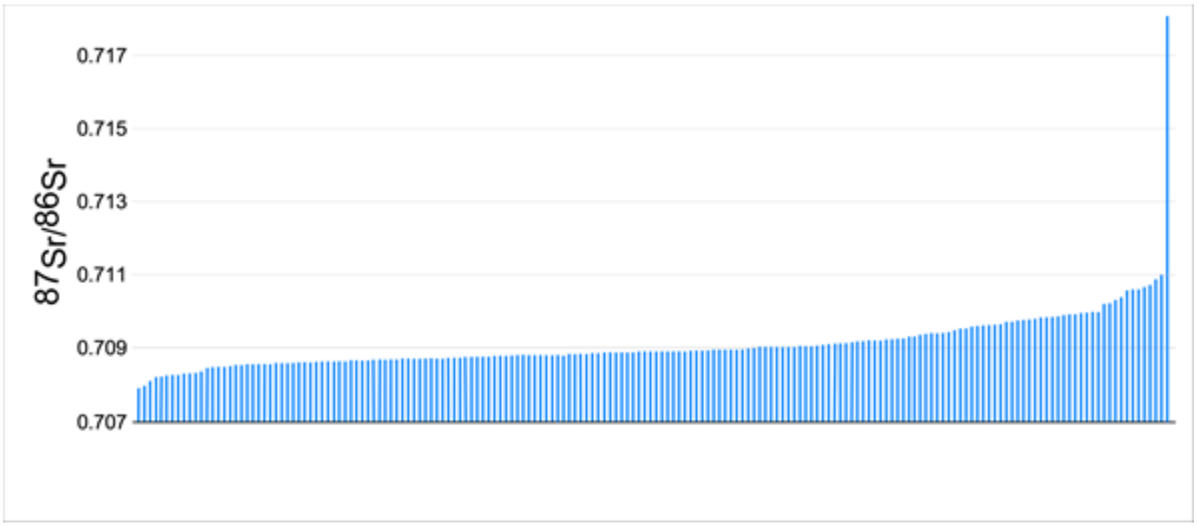

Supplementary Figure 6.3. Bar graph of ranked $^{87}Sr/^{86}Sr$ Values in human tooth enamel at Bury.

A histogram of these same values is shown in Fig. S5.6. The 11 outliers identified previously appear outside the narrow range from 0.7082 to 0.7106.

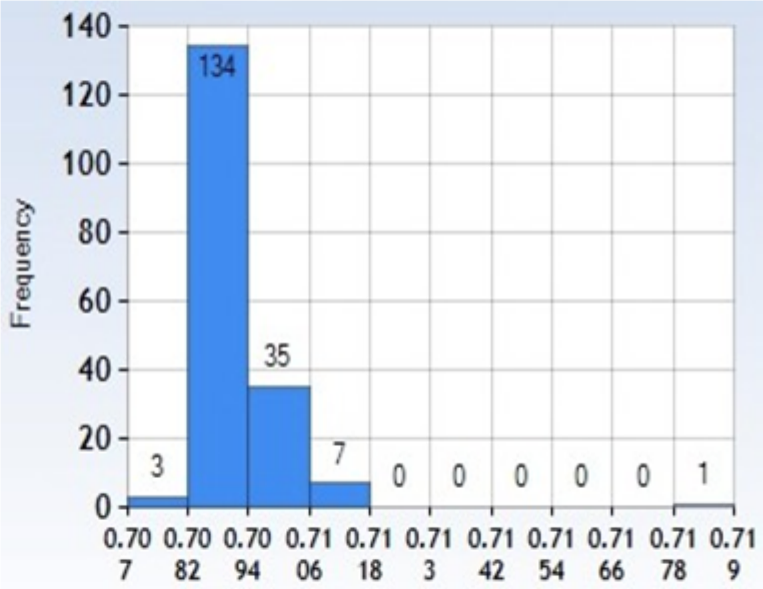

Supplementary Figure 6.4. Histogram of $^{87}Sr/^{86}Sr$ Values in human tooth enamel at Bury.

It is also possible to use box and whisker plots to identify outliers. A plot is shown in Fig. S5.7. The boundary for Q1 is 0.7087 and for Q3 it is 0.7093 and the median is 0.7089. The quartile analysis identified 11 individuals with values at 0.7102, 0.7103, 0.7104, 0.7106, 0.7106, 0.7106, 0.7107, 0.7108, 0.7109, 0.7110, 0.7181 —all at the higher end of the range. Low values are not considered. In fact the eyeball method of identifying changes in the curve of the ranked values is probably more accurate.

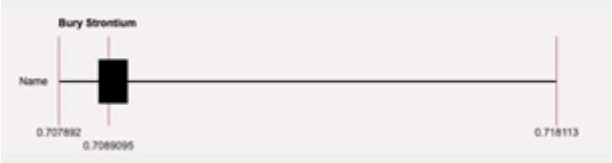

Supplementary Figure 6.5. Box and whisker plot of Bury $^{87}Sr/^{86}Sr$ data.

Oxygen isotopes (618O) were measured on 38 individuals with a mean of -4.0, 1 sd of 0.82, min of -7.99, and max -3.03. A plot of 618O vs. $^{87}Sr/^{86}Sr$ for these 38 samples is shown in Fig. S5.8. Oxygen isotopes range between -3.0 and -5.0 with one unusual exception at -8.0; that individual was identified as local in the analysis of the $^{87}Sr/^{86}Sr$ values. This value of 618O may be aberrant or real, but is very distant from the remainder of the samples.

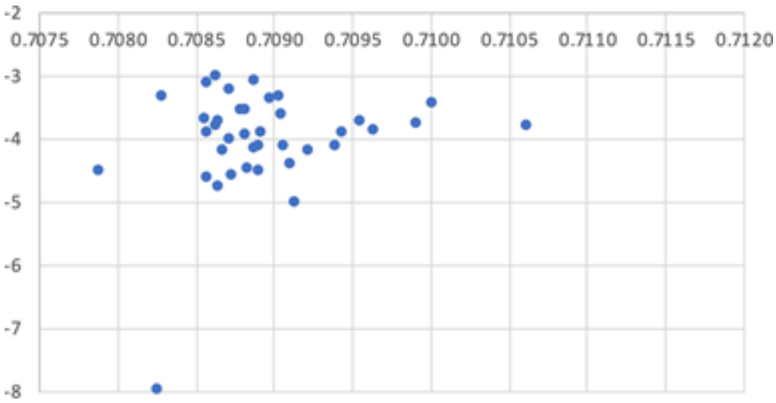

Supplementary Figure 6.5. Plot of 818O vs. $^{87}Sr/^{86}Sr$ for 38 samples.

It is important to keep in mind that the Paris Basin is a very large area with similar strontium isotope ratios so that individuals coming from outside this area would have to move a substantial distance. At the same time the differences between local and non-local in the samples investigated here is limited so that the non-local individuals identified here may be from within the Paris Basin and not at great distance from the local settlements.

Supplementary Note 6.4 - Summary and Conclusions

Isotopic proveniencing of collective burials has been done before in France. The burial mound of Le Tumulus des Sables in southwestern France dates from the Neolithic to the Iron Age[159]. Radiocarbon dating and isotopic analyses of human teeth was used to investigate the chronology, diet and mobility of the occupants. $^{87}Sr/^{86}Sr$ and 618O (on enamel) values were consistent with a local origin in the surrounding region, with one exception. These results documented the use of this burial mound by a local population over a period of about 2000 years.

In general terms, the results from both Le Tumulus des Sables and Bury suggest that there was limited human mobility in the late Neolithic. This pattern appears to be confirmed by the study of Goude et al. (2012)[160] who presented the first $^{87}Sr/^{86}Sr$ isotope results obtained on Neolithic human enamel from five Middle Neolithic (c. 4500–3500 cal BC) sites located in two geographic areas in Southern France. Their results did not support their hypothesis of mobility for a number of individuals initially considered as outsiders. Thus in contrast to the Early Neolithic in which movement seems a characteristic of life as farming populations expanded to occupy much of Europe, the later Neolithic appears to be more stable and sedentary.

It is clear that the isotopic proveniencing of human remains using strontium isotopes has been successful at Bury. Several individuals have been identified as non-local from outside the immediate area of the site. At the same time it is important to remember that the Paris Basin is a very large and generally homogeneous area geologically and isotopically so that some of the local individuals identified may well be from the larger region of the basin. It is also important to remember that isotopic proveniencing can only identify first generation immigrants so that evidence for these individuals is striking and important. In the future we hope to continue this study with additional analyses of carbon and oxygen isotopes in tooth enamel which may provide further new insights on the movement of people in prehistory.

Supplementary Information 7 - Burial positions within each pedigree at Bury

Philippe Chambon

Phase 1,
pedigree 1.A

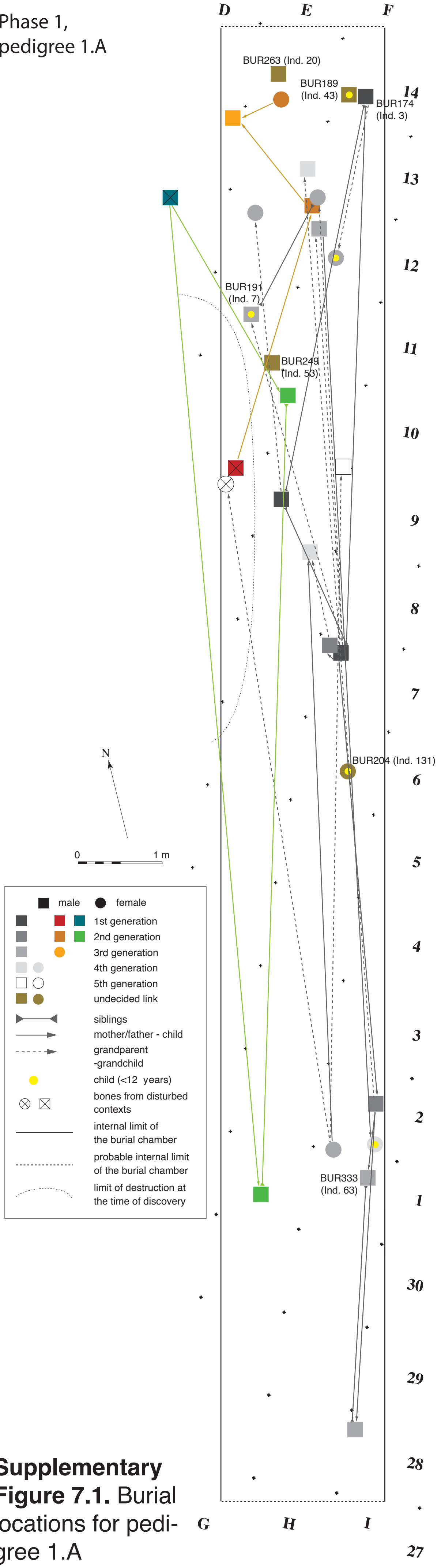

Supplementary Figure 7.1. Burial locations for pedigree 1.A

Phase 1,
pedigree 1.B

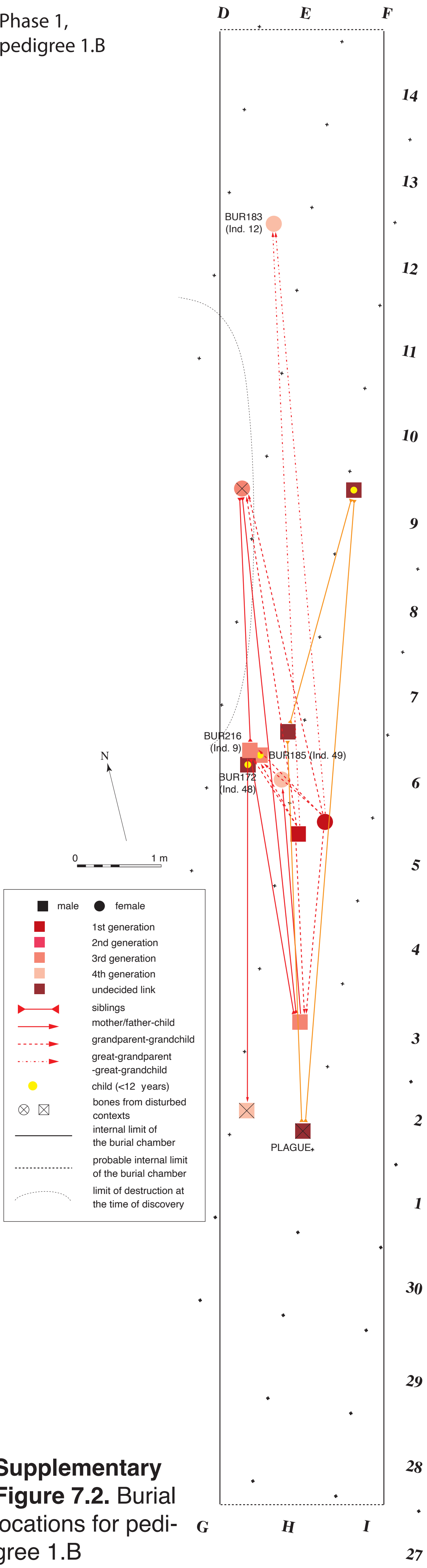

Supplementary Figure 7.2. Burial locations for pedigree 1.B

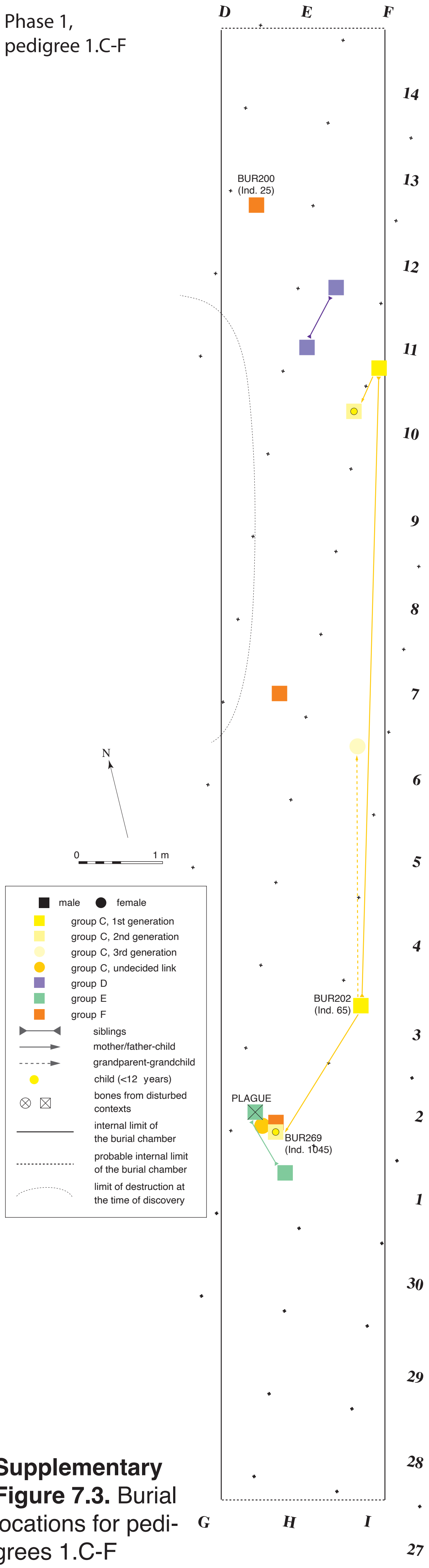

Supplementary Figure 7.3. Burial locations for pedigrees 1.C-F

Phase 1, Unrelated

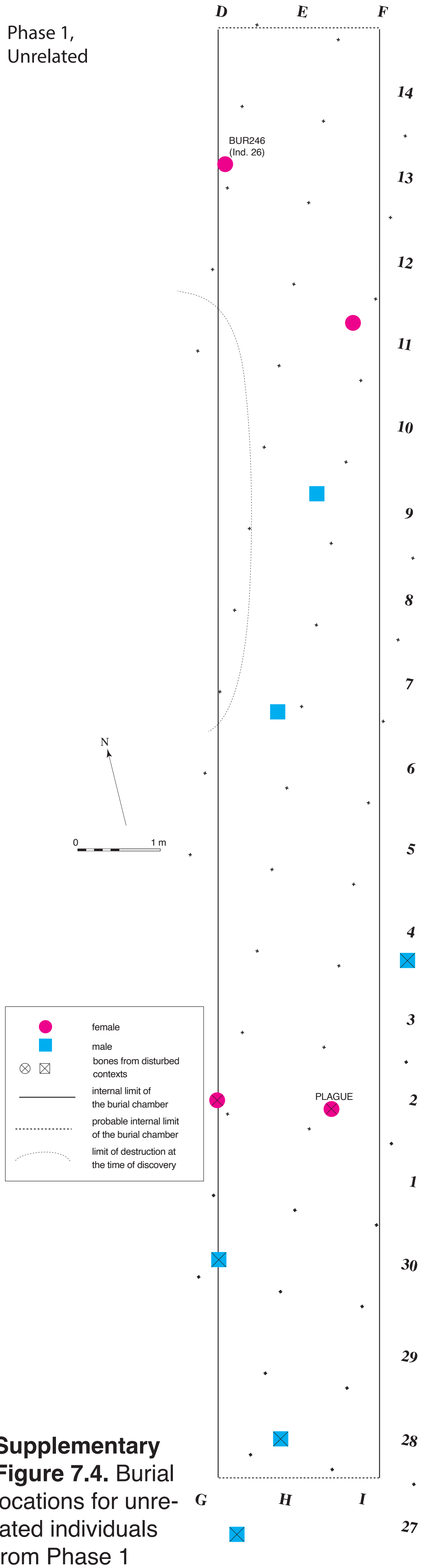

Supplementary Figure 7.4. Burial locations for unrelated individuals from Phase 1

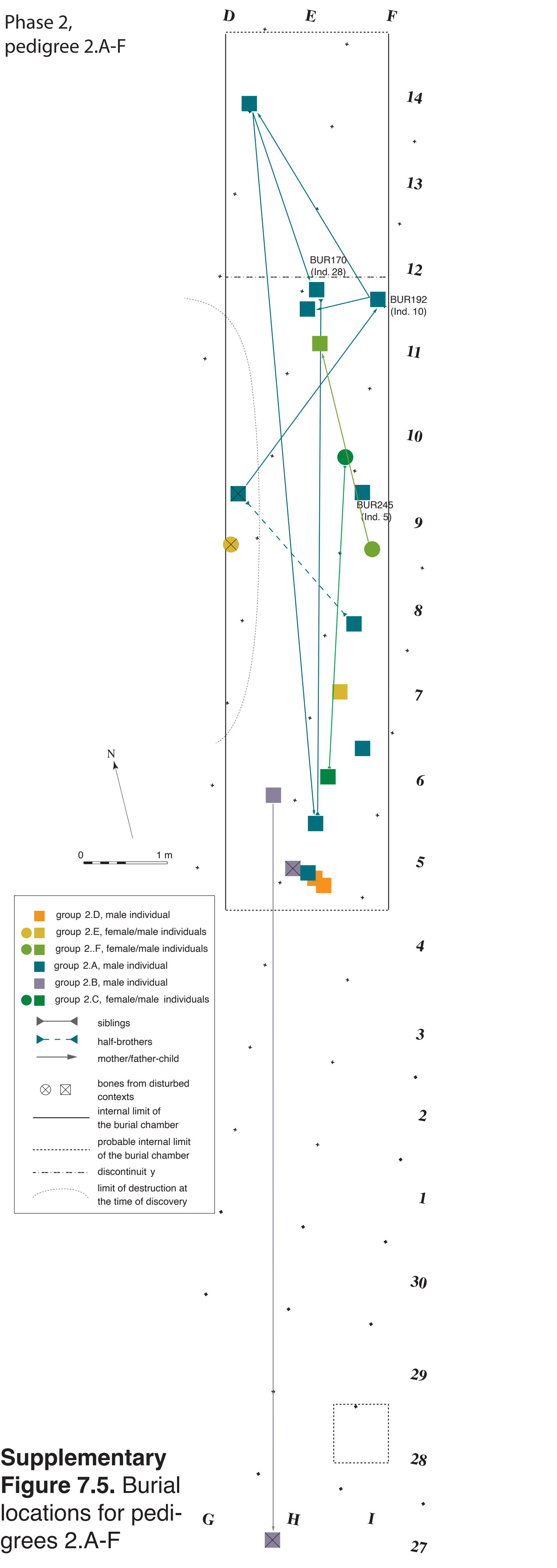

Supplementary Figure 7.5. Burial locations for pedigrees 2.A-F

Phase 2,
Unrelated

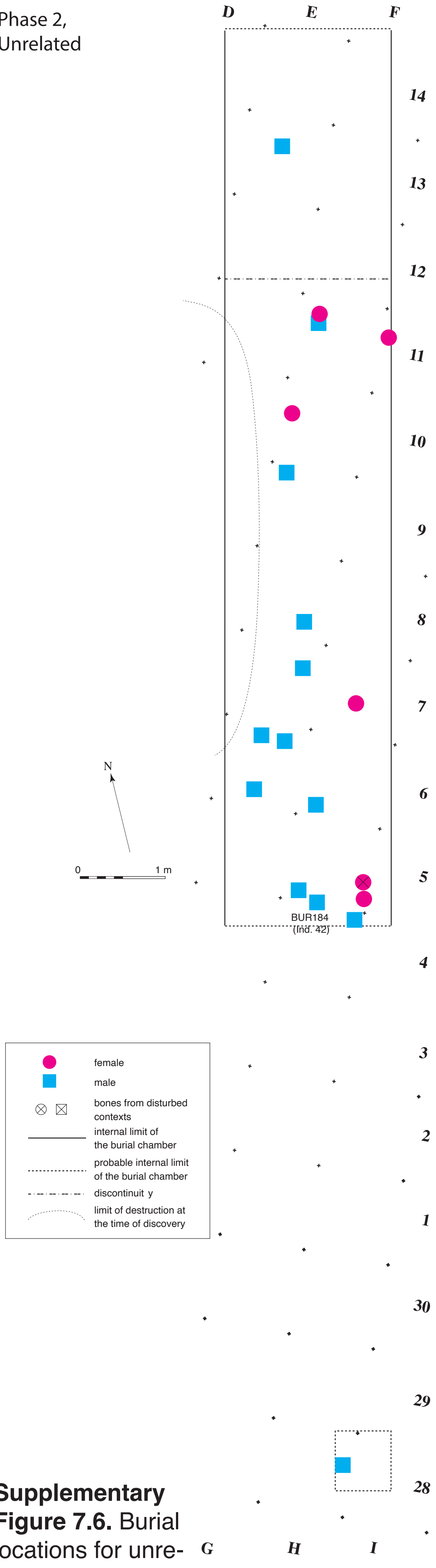

Supplementary Figure 7.6. Burial locations for unrelated individuals from Phase 2

References

82. Masset, C. Sur des anomalies d'ordre paléodémographique observées dans quelques sépultures néolithiques. in *IXe Congrès de l'UISPP, Thèmes spécialisés* 78–107 (Nice, 1976).
83. Ledermann, S. *Nouvelles Tables-Types de Mortalité*. (INED, Paris, 1969).
84. Sellier, P. La mise en évidence d'anomalies démographiques et leur interprétation: population, recrutement et pratiques funéraires du tumulus de Courtesoult. in *Nécropoles et société au premier âge du Fer: le tumulus de Courtesoult* (ed. Piningre, J.-F.) 188–202 (Maison des sciences de l'homme, Paris, 1996).
85. Blin, A. *Les Allées Sépulcrales Du Bassin Parisien à La Fin Du Néolithique: L'exemple de La Chaussée-Tirancourt*. (CNRS Éditions, Paris, 2022).
86. Bocquentin, F. La sépulture collective Bardouville. (Université Paris 1, Paris, 1998).
87. Marçais, A.-S. Des morts ensemble: étude du recrutement des inhumés dans les sépultures collectives dans le Bassin parisien à la fin du Néolithique. (Nanterre Université, 2016).
88. Bendezu-Sarmiento, J. Le Laris-Goguet à Feigneux (Oise), une grotte sépulcrale de la fin du Néolithique. De nouvelles données à partir d'une étude archéologique et anthropologique effectuée sur les sujets immatures. *Revue archéologique de Picardie 1999* 63–82 (1999).
89. Donat, R. Société, envrionnement et état sanitaire au Néolithique récent. Les groupes humains des hypogées I et II du Mont-Aimé. (Université Paul Sabatier, Toulouse, 2020).
90. Le Roy, M., de Becdelièvre, C., Rottier, S. & Thiol, S. De feu et d'os : la sépulture collective néolithique de la Truie Pendue (Passy-Veron, Yonne) Application SIG. *Préhistoires méditerr.* (2019) doi:10.4000/pm.1793.
91. Valentin, F. Variabilité humaine au Néolithique récent et final dans le Bassin parisien. *Gallia Préhistoire* **39**, 239–225 (1997).
92. Bach, H. & Bach, A. Anthropologische Analyse des Walternienburg/Bernburger Kollektivgrabes von Schönstedt im Thüringer Becken. *Alt-Thüringen* **12**, 59–107 (1972).
93. Feustel, R. & Ullrich, H. Totenhütten der neolitischen Walternienburger Gruppe. *Alt-Thüringen*

7, 105–202 (1965).

94. Meyer, C., Kranzbühler, J., Drings, S., Nehlich, O., Richards M. P., and Alt, K. W. Die Menschlichen Skelettfunde aus der neolitischen Totenhütte von Benzingerode. in *Die Totenhütte von Benzingerode* (ed. Berthold, B.) 107–146 (Landesmuseum für Vorgeschichte, Halle, 2008).
95. Browning, S. R. & Browning, B. L. Accurate non-parametric estimation of recent effective population size from segments of identity by descent. *Am. J. Hum. Genet.* **97**, 404–418 (2015).
96. Leroyer, C. *Homme, climat, végétation au Tardi-et Postglaciaire dans le Bassin parisien: apports de l'étude palynologique des fonds de vallée*. (Paris 1, 1997).
97. Leroyer, C. *Impacts Interculturels Au Néolithique Moyen. Du Terroir Au Territoire : Sociétés et Espaces*. (ARTEHIS Éditions, 2006).
98. Leroyer, C. & Allenet, G. L'anthropisation du paysage végétal d'après les données polliniques: l'exemple des fonds de vallées du Bassin parisien. in *L'érosion entre Société, Climat et Paléoenvironnement.* (ed. Lespez, P. A. et) 63–72 (2006).
99. David, R. *Modélisation de la végétation holocène du Nord-Ouest de la France: Reconstruction de la chronologie et de l'évolution du couvert végétal du Bassin parisien et du Massif armoricain*. (Université de Rennes, 2014).
100. Sugita, S. Theory of quantitative reconstruction of vegetation I: pollen from large sites REVEALS regional vegetation composition. *Holocene* **17**, 229–241 (2007).
101. Mercuri, A. M. *et al.* From influence to impact: The multifunctional land use in Mediterranean prehistory emerging from palynology of archaeological sites (8.0-2.8 ka BP). *Holocene* **29**, 830–846 (2019).
102. Berglund, B. E. The cultural landscape during 6000 years in southern Sweden–the ystad project. *Ecological Bulletins* **41**, (1991).
103. Gaillard, M.J., Berglund BE, Göransson H, Hjelmroos M, Kolstrup E and Regnéll J. Chronology of the pollen diagrams from the Ystad area. in *The Cultural Landscape During 6000 Years in*

Southern Sweden: The Ystad Project (ed. Berglund, B. E.) 489–495 (Ecological Bulletins, 1991).

104. Håkansson, H. & Regnéll, J. Diatom succession related to land use during the last 6000 years: a study of a small eutrophic lake in southern Sweden. *J. Paleolimnol.* **8**, (1993).
105. Githumbi, E. *et al.* European pollen-based REVEALS land-cover reconstructions for the Holocene: methodology, mapping and potentials. *Earth Syst. Sci. Data* **14**, 1581–1619 (2022).
106. Trondman, A.-K. *et al.* Pollen-based quantitative reconstructions of Holocene regional vegetation cover (plant-functional types and land-cover types) in Europe suitable for climate modelling. *Glob. Chang. Biol.* **21**, 676–697 (2015).
107. Abraham, V., Oušková, V. & Kuneš, P. Present-day vegetation helps quantifying past land cover in selected regions of the Czech Republic. *PLoS One* **9**, e100117 (2014).
108. Crema, E. R. & Bevan, A. Inference from large sets of radiocarbon dates: Software and methods. *Radiocarbon* **63**, 23–39 (2021).
109. Lechterbeck, J. *et al.* Is Neolithic land use correlated with demography? An evaluation of pollen-derived land cover and radiocarbon-inferred demographic change from Central Europe. *Holocene* **24**, 1297–1307 (2014).
110. Woodbridge, J. *et al.* The impact of the Neolithic agricultural transition in Britain: a comparison of pollen-based land-cover and archaeological 14C date-inferred population change. *J. Archaeol. Sci.* **51**, 216–224 (2014).
111. Bevan, A. *et al.* Holocene fluctuations in human population demonstrate repeated links to food production and climate. *Proc. Natl. Acad. Sci. U. S. A.* **114**, E10524–E10531 (2017).
112. Theuerkauf, M., Couwenberg, J., Kuparinen, A. & Liebscher, V. A matter of dispersal: REVEALSinR introduces state-of-the-art dispersal models to quantitative vegetation reconstruction. *Veg. Hist. Archaeobot.* **25**, 541–553 (2016).
113. Wickham, H., François, R., Henry, L., Müller, K. & Vaughan, D. *dplyr: A Grammar of Data Manipulation. Rpackage Version 1.1.0*. (2023).
114. Behre, K.-E. The Interpretation of Anthropogenic Indicators in Pollen Diagrams. *Pollen et Spores*

23, 225–245 (1981).

115. Behre, K.-E. *Anthropogenic Indicators in Pollen Diagrams*. (Routledge, 1986).
116. Feeser, I. & Dörfler, W. The Early Neolithic in pollen diagrams from eastern Schleswig-Holstein and western Mecklenburg - evidence for a 1000 years cultural adaptive cycle? in *The Dąbki Site in Pomerania and the Neolithisation of the North European Lowlands (c. 5000–3000 calBC)* (eds. Kabaciński, J., Hartz, S., Raemaekers, D. C. M. & Terberger, T.) (Verlag Marie Leidorf GmbH, Schleswig-Holstein and Western Mecklenburg).
117. Iversen, J. Landnam i Danmarks Stenalder. En pollenanalytisk Undersøgelse over det første Landbrugs Indvirkning paa Vegetationsudviklingen. *Danmarks Geologiske Undersøgelse II.* **66**, 1–68 (1941).
118. Kirleis, W. & Fischer, E. Neolithic cultivation of tetraploid free threshing wheat in Denmark and Northern Germany: implications for crop diversity and societal dynamics of the Funnel Beaker Culture. *Veg. Hist. Archaeobot.* **23**, 81–96 (2014).
119. Odgaard, B. V. & Rasmussen, P. Origin and temporal development of macro-scale vegetation patterns in the cultural landscape of Denmark. *J. Ecol.* **88**, 733–748 (2000).
120. Rasmussen, P. Mid-to late-Holocene land-use change and lake development at Dallund S0, Denmark: vegetation and land-use history inferred from pollen data. *Holocene* **15**, 1116–1129 (2005).
121. Rasmussen, P. & Bradshaw, E. G. Mid-to late-Holocene land-use change and lake development at Dallund S0, Denmark: study aims, natural and cultural setting, chronology and soil erosion history. *Holocene* **15**, 1105–1115 (2005).
122. Ebbesen, K. *Nordjyske gravkister med indgang. Bøstrup-kisterne*. *Aarbøger for Nordisk Oldkyndighed og historie* **1983**, 5–65 (1985).
123. Fabech, C. Storstenskisten fra Blære. *Kuml 1986* 45–75 (1986).
124. Hübner, E. Jungneolitische Gräber auf der Jütischen Halbinsel. Typologische und chronologische Studien zur Einzelgrabkultur. *Nordiske Fortidsminder Serie B* **24**, (2005).

125. Liversage, D. En hellekiste ved Gerdrup, Københavns amt. *Aarbøger for Nordisk Oldkyndighed og historie* **1964**, 32–62 (1965).

126. Sterum, N. Hellekister i sen jysk enkeltgravskultur. Fra periferien af jyske enkeltgravshøje. *Aarbøger for Nordisk Oldkyndighed og historie* **1974**, 61–103 (1976).

127. Dehn, T., Hansen, S. I. and Westphal, J. Restoration of megalithic tombs in Denmark. in *Counterpoint: Essays in Archaeology and Heritage Studies in Honour of Professor Kristian Kristiansen* 695–702 (BAR International Series 2508, 2013).

128. Hansen, S. I. *Jættestuebyggerne. Arkitektur I Danmarks Stenalder*. (Kahrius, 2016).

129. Dehn, T. & Hansen, S. I. Birch bark in Danish passage graves. *J. Dan. Archaeol.* **14**, 23–44 (2006).

130. Price, T. D., Schoeninger, M. J. & Armelagos, G. J. Bone chemistry and past behavior: an overview. *J. Hum. Evol.* **14**, 419–447 (1985).

131. Sillen, A. & Kavanagh, M. Strontium and paleodietary research: A review. *Am. J. Phys. Anthropol.* **25**, 67–90 (1982).

132. Price, T. D. An introduction to the isotopic studies of ancient human remains. *J. N. Atl.* **7**, 71–87 (2015).

133. Montgomery, J. Passports from the past: Investigating human dispersals using strontium isotope analysis of tooth enamel. *Ann. Hum. Biol.* **37**, 325–346 (2010).

134. Price, T. D., C. Johnson, J. Ezzo, J. Burton, and J. Ericson. Residential mobility in the American Southwest: A preliminary study using strontium isotope analysis. *Journal of Archaeological Science* **21**, 315–330 (1994).

135. Teresa M. Mensing, G. F. *Isotopes: Principles and Applications*. (Wiley, New York, 2004).

136. Price, T. D., Burton, J. H. & Bentley, R. A. The characterization of biologically available strontium isotope ratios for the study of prehistoric migration. *Archaeometry* **44**, 117–135 (2002).

137. Sillen, A., Hall, G., Richardson, S. & Armstrong, R. 87Sr/86Sr ratios in modern and fossil food-webs of the Sterkfontein Valley: implications for early hominid habitat preference. *Geochim. Cosmochim. Acta* **62**, 2463–2473 (1998).

138. Antoine, P., Catt, J., Lautridou, J.-P. & Sommé, J. The loess and coversands of northern France and southern England. *J. Quat. Sci.* **18**, 309–318 (2003).

139. Price. T. Douglas, Robert Frei, Ute Brinker, Gundula Lidke, Karin Frei, Thomas Terberger, and Detlef Jantzen. Multi-Isotope Analysis of Human Remains from a Bronze Age Battlefield in Northeast Germany. *Archaeological and Anthropological Sciences* (2017) doi:10.1007/s12520-017-0529-y.

140. Maréchal, J.-C. & Rouillard, J. Groundwater in France: Resources, Use and Management Issues. in *Sustainable Groundwater Management: A Comparative Analysis of French and Australian Policies and Implications to Other Countries* (ed. J.-D. Rinaudo, C. Holley, S. Barnett, & M. Montginoul) 17–45 (Springer International Publishing, 2020).

141. Jones, C. E. Seawater strontium isotopes, oceanic anoxic events, and seafloor hydrothermal activity in the Jurassic and Cretaceous. *Am. J. Sci.* **301**, 112–149 (2001).

142. Vezier, J. Strontium isotopes in seawater through time. *Annual Reviews: Earth and Planetary Science* **17**, 141–167 (1989).

143. Vezier, J. *et al.* 87Sr/86Sr, б13C and б18O evolution of Phanerozoic seawater. *Chemical Geology* **16**, 59–88 (1999).

144. Willmes, M. *et al.* Mapping of bioavailable strontium isotope ratios in France for archaeological provenance studies. *Applied Geochemistry* **90**, 75–86 (2018).

145. Rousseau, D.-D. *et al.* European glacial dust deposits: Geochemical constraints on atmospheric dust cycle modeling. *Geophys. Res. Lett.* **41**, 7666–7674 (2014).

146. Willmes, M., Grün, R. & Mcmorrow, L. Strontium isotope ratios of plant and soil samples from France. *Research School of Earth Sciences* (2013).

147. Willmes, M. *et al.* The IRHUM (Isotopic Reconstruction of Human Migration) database – bioavailable strontium isotope ratios for geochemical fingerprinting in France. *Earth Syst. Sci. Data* **6**, 117–122 (2014).

148. Evans, J. A., Chenery, C. A. & Montgomery, J. A summary of strontium and oxygen isotope

variation in archaeological human tooth enamel excavated from Britain. *J. Anal. At. Spectrom.* **27**, 754 (2012).

149. White, C. D., Spence, M. W., Longstaffe, F. J., Stuart-Williams, H. & Law, K. R. Geographic identities of the sacrificial victims from the Feathered Serpent Pyramid, Teotihuacan: Implications for the nature of state power. *Lat. Am. Antiq.* **13**, 217–236 (2002).
150. Luz, B. & Kolodny, Y. Oxygen isotope variations in phosphate of biogenic apatites, IV. Mammal teeth and bones. *Earth Planet. Sci. Lett.* **75**, 29–36 (1985).
151. Luz, B., Kolodny, Y. & Horowitz, M. Fractionation of oxygen isotopes between mammalian bone-phosphate and environmental drinking water. *Geochim. Cosmochim. Acta* **48**, 1689–1693 (1984).
152. Lachniet, M. S. & Patterson, W. P. Oxygen isotope values of precipitation and surface waters in northern Central America (Belize and Guatemala) are dominated by temperature and amount effects. *Earth Planet. Sci. Lett.* **284**, 435–446 (2009).
153. White, C. D., Longstaffe, F. J. & Law, K. R. Revisiting the Teotihuacan connection at Altun Ha: Oxygen-isotope analysis of tomb F-8/1. *Anc. Mesoam.* **12**, 65–72 (2001).
154. White, C. D., Spence, M. W., Longstaffe, F. J. & Law, K. R. Demography and ethnic continuity in the Tlailotlacan enclave of Teotihuacan: the evidence from stable oxygen isotopes. *J. Anthropol. Archaeol.* **23**, 385–403 (2004).
155. White, C. D., Spence, M. W., Longstaffe, F. J. & Law, K. R. Testing the nature of teotihuacán imperialism at kaminaljuyú using phosphate oxygen-isotope ratios. *J. Anthropol. Res.* **56**, 535–558 (2000).
156. White, C. D., Spence, M. W., Le Q. Stuart-Williams, H. & Schwarcz, H. P. Oxygen isotopes and the identification of geographical origins: The valley of Oaxaca versus the valley of Mexico. *J. Archaeol. Sci.* **25**, 643–655 (1998).
157. Wright, L. E. & Schwarcz, H. P. Stable carbon and oxygen isotopes in human tooth enamel: identifying breastfeeding and weaning in prehistory. *Am. J. Phys. Anthropol.* **106**, 1–18 (1998).

158. White, C. D., Storey, R., Longstaffe, F. J. & Spence, M. W. Immigration, assimilation, and status in the ancient city of Teotihuacan: Stable isotopic evidence from Tlajinga 33. *Lat. Am. Antiq.* **15**, 176–198 (2004).

159. James, H. F. *et al.* Who’s been using my burial mound? Radiocarbon dating and isotopic tracing of human diet and mobility at the collective burial site, Le Tumulus des Sables, southwest France. *J. Archaeol. Sci. Rep.* **24**, 955–966 (2019).

160. Goude, G., Castorina, F., Herrscher, E., Cabut, S. & Tafuri, M. A. First strontium isotope evidence of mobility in the Neolithic of Southern France. *Eur. J. Archaeol.* **15**, 421–439 (2012).
