# Supplementary material for: Population discontinuity in the Paris Basin linked to evidence of the Neolithic decline
Source: Nat Ecol Evol. 2026 Apr 3;10(4):677–88. doi: 10.1038/s41559-026-03027-z (PMC13076197; doi:10.1038/s41559-026-03027-z)
Supplement: Supplementary file 2 — Reporting Summary [file 41559_2026_3027_MOESM2_ESM.pdf]

nature portfolio

Corresponding author(s): Martin Sikora & Eske Willerslev

Last updated by author(s): Jan 23, 2026

Reporting Summary

Nature Portfolio wishes to improve the reproducibility of the work that we publish. This form provides structure for consistency and transparency in reporting. For further information on Nature Portfolio policies, see our Editorial Policies and the Editorial Policy Checklist.

Statistics

For all statistical analyses, confirm that the following items are present in the figure legend, table legend, main text, or Methods section.

n/a	Confirmed	
☐	☒	The exact sample size (*n*) for each experimental group/condition, given as a discrete number and unit of measurement
☐	☒	A statement on whether measurements were taken from distinct samples or whether the same sample was measured repeatedly
☐	☒	The statistical test(s) used AND whether they are one- or two-sided<br>*Only common tests should be described solely by name; describe more complex techniques in the Methods section.*
☒	☐	A description of all covariates tested
☒	☐	A description of any assumptions or corrections, such as tests of normality and adjustment for multiple comparisons
☐	☒	A full description of the statistical parameters including central tendency (e.g. means) or other basic estimates (e.g. regression coefficient) AND variation (e.g. standard deviation) or associated estimates of uncertainty (e.g. confidence intervals)
☐	☒	For null hypothesis testing, the test statistic (e.g. *F*, *t*, *r*) with confidence intervals, effect sizes, degrees of freedom and *P* value noted<br>*Give P values as exact values whenever suitable.*
☒	☐	For Bayesian analysis, information on the choice of priors and Markov chain Monte Carlo settings
☒	☐	For hierarchical and complex designs, identification of the appropriate level for tests and full reporting of outcomes
☒	☐	Estimates of effect sizes (e.g. Cohen's *d*, Pearson's *r*), indicating how they were calculated

Our web collection on statistics for biologists contains articles on many of the points above.

Software and code

Policy information about availability of computer code

Data collection: Illumina NovaSeq system

Data analysis:
ANGSD (0.931)
Bcftools (1.16)
bedtools (v2.31.0)
bwa (0.7.17)
convertf(version: 5722)
dates (Version 4010)
decluster
EPA-ng v0.3.8
Gappa (v0.8.0)
GATK (v4.3.0.0)
gcta64 (v1.94.1)
glimpse (v1.1.1)
preseq (v 3.2.0)
ibdseq (r1206)
picard (v 3.1.1)
HAPLOGREP (2.1.25)
MUTSERVE (1.3.0)
Jvarkit (v dbdbed3a9)
Java (v 17.0.3)

KIN (0.1.0)
KINgaroo (0.1.0)
King (v 2.3.0)
Krakenuniq (v 1.0.4)
mapDamage (2.2.0-86-g81d0aca)
METADMG (v 0.2-86-gcba5d46)
Mosdepth (v 0.3.3)
ngsRelate
plink (v1.90b6.21)
ngsngs (v0.9.0)
python (3.10.8)
PRIMUS (v1.9.0)
msPrime (1.2.0)
READ2.py (v2.00)
raxml-ng (v. 1.2.0)
realSFS
samtools (v 1.21)
seqtk (1.3-r106)
smartpca (eigensoft v. 8.0.0)

R packages:
Argparse (v 2.2.2)
data.table (v 1.14.8)
doParallel (1.0.17)
dplyr (1.1.1)
forcats (v 1.0.0)
foreach (v1.5.2)
furrr (v 0.3.1)
ggplot2 (v3.4.2)
ggtree (v3.6.2)
ggraph (v 2.1.0)
ggVennDiagram (v 1.5.0)
gridExtra (v 2.3)
igraph (v 1.4.2)
plyr (v 1.8.8)
purrr (v 1.0.1)
readr (v 2.1.4)
scales (v 1.4.0)
stringr (v 1.5.0)
tidyverse (v 2.0.0)
tidygraph (v 1.2.3)
vcfR (v 1.14.0)

For manuscripts utilizing custom algorithms or software that are central to the research but not yet described in published literature, software must be made available to editors and reviewers. We strongly encourage code deposition in a community repository (e.g. GitHub). See the Nature Portfolio guidelines for submitting code & software for further information.

Data

Policy information about availability of data

All manuscripts must include a data availability statement. This statement should provide the following information, where applicable:

- Accession codes, unique identifiers, or web links for publicly available datasets
- A description of any restrictions on data availability
- For clinical datasets or third party data, please ensure that the statement adheres to our policy

Fastq files with collapsed and adapter trimmed reads from this study have been deposited in the European Nucleotide Archive under accession number PRJEB95770 (Supplementary Table 12).

Research involving human participants, their data, or biological material

Policy information about studies with human participants or human data. See also policy information about sex, gender (identity/presentation), and sexual orientation and race, ethnicity and racism.

Reporting on sex and gender	Not appllicable
Reporting on race, ethnicity, or other socially relevant groupings	Not appllicable
Population characteristics	Not appllicable
Recruitment	Not appllicable

Ethics oversight	Not appllicable

Note that full information on the approval of the study protocol must also be provided in the manuscript.

Field-specific reporting

Please select the one below that is the best fit for your research. If you are not sure, read the appropriate sections before making your selection.

☒ Life sciences ☐ Behavioural & social sciences ☐ Ecological, evolutionary & environmental sciences

For a reference copy of the document with all sections, see nature.com/documents/nr-reporting-summary-flat.pdf

Life sciences study design

All studies must disclose on these points even when the disclosure is negative.

Sample size	No tests were carried out to predetermine sample size. Sample size was determined by the availability of archaeological material, and on the DNA preservation in these samples.
Data exclusions	Samples with a final depth of coverage under 0.01X or libraries with high contamination estimates as determined by ContamMix (v1.0.10) were excluded from downstream analyses. This cutoff was predetermined and follows Seersholm et al. 2024
Replication	Out of the 181 samples analysed in this study, 145 are represented by more than one sequencing library. Having multiple sequencing libraries for each sample serves to validate sequencing results and to pinpoint potential sample swaps. Of the 145 samples represented by more than one sequencing library, 82 samples have multiple sequencing libraries of sufficient coverage to assess whether these belong to the same genetic individual (>0.01). Except from one library pair, all of these where characterised as coming from the same individual using READ2. The library pair not characterised as coming from the same individual, was characterised as "first degree relatives", presumably due to the low coverage of both libraries (0.01X and 0.01X). Apart from this type of replication, replication of experimental findings is generally not applicable for this kind of ancient DNA study because of the unique nature of ancient human remains.
Randomization	Not relevant. Sample allocation was not random, but followed archaeological burial phase and biological kinship.
Blinding	Not applicable. Ancient DNA research is observational, with samples defined by archaeological context rather than experimental assignment, and all laboratory and bioinformatic procedures were applied uniformly using predefined protocols. As there were no interventions or subjective outcome assessments, blinding was not relevant to data collection or analysis.

Reporting for specific materials, systems and methods

We require information from authors about some types of materials, experimental systems and methods used in many studies. Here, indicate whether each material, system or method listed is relevant to your study. If you are not sure if a list item applies to your research, read the appropriate section before selecting a response.

Materials & experimental systems

n/a	Involved in the study	
☒	☐	Antibodies
☒	☐	Eukaryotic cell lines
☐	☒	Palaeontology and archaeology
☒	☐	Animals and other organisms
☒	☐	Clinical data
☒	☐	Dual use research of concern
☒	☐	Plants

Methods

n/a	Involved in the study	
☒	☐	ChIP-seq
☒	☐	Flow cytometry
☒	☐	MRI-based neuroimaging

Palaeontology and Archaeology

Specimen provenance	The sampling was made in 2013 under the supervision of Laure Salanova, the scientific authority of the Bury project. In total, we collected 181 ancient human teeth samples, representing 179 individuals (57% of the total of 316 individuals estimated to have been buried at the site). We decided to sample mandibular teeth, since the skulls could not be linked to the identified archaeological individuals (Supplementary table 1). In total, we sampled more than 71% of the testable mandibles, including all but one of the mandibles from identified archaeological individuals. Lastly, we also sampled 21 loose teeth to ensure the broadest representation possible. Along with all other samples, these loose teeth were subsequently screened genetically to identify any duplicates.
Specimen deposition	Leftover DNA digests, extract and sequencing libraries are stored at the DNA laboratory facilities at Globe Institute, Copenhagen. Upon completion of this project, leftover bone material will be returned to respective museum or university collections from which they were sampled.

Dating methods

Radiocarbon dating was performed at the Keck carbon cycle AMS facility, University of California, Irving. The samples were decalcified in 1N HCl, gelatinized at 60°C and pH 2, and ultrafiltered to select a high molecular wt fraction (>30kDa). δ13C and δ15N values were measured to a precision of <0.1‰ and <0.2‰, respectively, on aliquots of ultrafiltered collagen, using a Fisons NA1500NC elemental analyzer/Finnigan Delta Plus isotope ratio mass spectrometer. Datings were calibrated in Oxcal 4.4.4 using the Intcal20 calibration curve.

☒ Tick this box to confirm that the raw and calibrated dates are available in the paper or in Supplementary Information.

Ethics oversight

No ethical approval was required for this study.

Note that full information on the approval of the study protocol must also be provided in the manuscript.

Plants

Seed stocks

Report on the source of all seed stocks or other plant material used. If applicable, state the seed stock centre and catalogue number. If plant specimens were collected from the field, describe the collection location, date and sampling procedures.

Novel plant genotypes

Describe the methods by which all novel plant genotypes were produced. This includes those generated by transgenic approaches, gene editing, chemical/radiation-based mutagenesis and hybridization. For transgenic lines, describe the transformation method, the number of independent lines analyzed and the generation upon which experiments were performed. For gene-edited lines, describe the editor used, the endogenous sequence targeted for editing, the targeting guide RNA sequence (if applicable) and how the editor was applied.

Authentication

Describe any authentication procedures for each seed stock used or novel genotype generated. Describe any experiments used to assess the effect of a mutation and, where applicable, how potential secondary effects (e.g. second site T-DNA insertions, mosiacism, off-target gene editing) were examined.
